# Molecular Mechanism of Mutant p53 Stabilization: The Role of HSP70 and MDM2

**DOI:** 10.1371/journal.pone.0051426

**Published:** 2012-12-12

**Authors:** Milena Wiech, Maciej B. Olszewski, Zuzanna Tracz-Gaszewska, Bartosz Wawrzynow, Maciej Zylicz, Alicja Zylicz

**Affiliations:** 1 Department of Molecular Biology, International Institute of Molecular and Cell Biology in Warsaw, Warsaw, Poland; 2 The Nencki Institute of Experimental Biology, PAS, Warsaw, Poland; 3 The Institute of Biochemistry and Biophysics, PAS, Warsaw, Poland; Semmelweis University, Hungary

## Abstract

Numerous p53 missense mutations possess gain-of-function activities. Studies in mouse models have demonstrated that the stabilization of p53 R172H (R175H in human) mutant protein, by currently unknown factors, is a prerequisite for its oncogenic gain-of-function phenotype such as tumour progression and metastasis. Here we show that MDM2-dependent ubiquitination and degradation of p53 R175H mutant protein in mouse embryonic fibroblasts is partially inhibited by increasing concentration of heat shock protein 70 (HSP70/HSPA1-A). These phenomena correlate well with the appearance of HSP70-dependent folding intermediates in the form of dynamic cytoplasmic spots containing aggregate-prone p53 R175H and several molecular chaperones. We propose that a transient but recurrent interaction with HSP70 may lead to an increase in mutant p53 protein half-life. In the presence of MDM2 these pseudoaggregates can form stable amyloid-like structures, which occasionally merge into an aggresome. Interestingly, formation of folding intermediates is not observed in the presence of HSC70/HSPA8, the dominant-negative K71S variant of HSP70 or HSP70 inhibitor. In cancer cells, where endogenous HSP70 levels are already elevated, mutant p53 protein forms nuclear aggregates without the addition of exogenous HSP70. Aggregates containing p53 are also visible under conditions where p53 is partially unfolded: 37°C for temperature-sensitive variant p53 V143A and 42°C for wild-type p53. Refolding kinetics of p53 indicate that HSP70 causes transient exposure of p53 aggregate-prone domain(s). We propose that formation of HSP70- and MDM2-dependent protein coaggregates in tumours with high levels of these two proteins could be one of the mechanisms by which mutant p53 is stabilized. Moreover, sequestration of p73 tumour suppressor protein by these nuclear aggregates may lead to gain-of-function phenotypes.

## Introduction

The p53 protein is a key human tumour suppressor. Its activity is mostly achieved by transcription factor potential modulating growth arrest, senescence and apoptosis as well as inhibition of several tumour protective factors (review by [1]). Under normal conditions the abundance and activity of p53 are governed through precise control of its regulatory network [2]–[6]. Various stressors such as DNA damage, heat shock, hypoxia and/or protooncogene activation lead to the increase in p53 levels and activity. Mutations in *TP53* tumour suppressor gene were identified in most human cancers [7]. More than 70% of these mutations are missense [8], [9]. Inactivating missense mutations of *TP53* are advantageous during cancer development due to their action as trans-dominant inhibitors of wild-type p53. Moreover, accumulation of point-mutated p53 protein in the cancer cell contributes to transformation and metastasis [10]. In this case, mutated p53 protein gains new pro-oncogenic functions [11], [12]. Molecular mechanisms underlying the gain-of-function phenotype, leading to increased cell migration and invasion, are still not clear. Several laboratories presented evidence that mutant p53 can be a transcription factor in its own right and that it can interfere with or modify functions of other proteins, with these scenarios not being mutually exclusive [13]–[18]. Mutated forms of p53 interact with their paralogs - p63 and p73, negatively regulating their function [11], [19]–[21]. Recently it was shown that structurally destabilized p53 mutants (e.g. p53 R175H) co-aggregate with p63 and p73 [22].

Several clinical studies suggest that elevated levels of mutated p53 correlate with more aggressive tumour progression and poor prognosis [23]–[26]. Similarly, *in vivo* studies employing knock-in mice in which either one or both p53 alleles were substituted by the mouse equivalent (R172H) of human p53 R175H, support the notion that the accumulation of mutant p53 contributes to transformation and metastasis.

An observation that partly indicates to the nature of mutant p53 pro-oncogenic function modifiers, is the fact that transgenic *Hsf1-*knockout, *Trp53 ^R172H^* mice do not develop cancer [27]. HSF1 transcription factor broadly controls heat shock protein synthesis. This suggests that heat shock proteins may contribute to mutant p53 stabilization and tumour progression. Mutated p53 R175H was indeed found to form a stable complex with HSP40, HSP70, HOP and HSP90 [28], [29]. Elevated levels of one or more major heat shock protein classes (e.g., HSP90, HSP70, HSP60, HSP40, HSP27) have been documented in many types of cancers [30]. In breast cancers, HSP70 overproduction correlates with bad prognosis [31]–[33]. Moreover, overexpression of HSP70 induces cellular transformation [34], [35].

The HSP70 (HSPA) protein family is evolutionary conserved and belongs to a class of molecular chaperones that are involved in housekeeping functions i.e. protein folding, translocation [36], [37], and degradation [38]–[41]. Under stress conditions HSPA proteins play multiple roles in proteostasis maintenance, such as prevention of protein aggregation and dissociation of existing aggregates [42]–[46], refolding of stress-damaged proteins [47], native protein conformation and activity maintenance [48], and inhibition of apoptosis [49].

HSP70 (HSPA1-A) possesses a weak ATPase activity, stimulated by protein substrates [50] and co-chaperones [51]. ATP hydrolysis induces HSP70 conformation flux that increases its affinity towards the substrate; the subsequent exchange of ADP for ATP induces conformational changes within HSP70 that trigger the release of the substrate [52]. Two possible modes of chaperone action have been identified. The passive (holdase) mode is based on the formation of HSP70/protein substrate complex, which in effect protects the substrate from conformational changes and/or aggregation [37]. Thus, the binding of ATP by HSP70 would induce the low-affinity conformation and partially inhibit the passive chaperone activity of HSP70. On the other hand, in the active (unfoldase) mode, repeated HSP70 binding and dissociation from the protein substrate can result in its conformational remodelling, leading to the dissociation of already existing aggregates or refolding of stress-damaged proteins [29]. As predicted by H. Pelham (1986) [53], this cyclic reaction requires ATP hydrolysis. The HSP70 K71S mutant employed in this study belongs to a class of the HSP70 ATPase nucleotide binding domain mutants. It behaves like WT HSP70 in an ADP-bound, high substrate affinity conformation. In other words, the K71S mutation “freezes” HSP70 in a biologically relevant conformation.

Heat shock protein family members actively participate in protein degradation. This is achieved through a close cooperation of the molecular chaperone with chaperone-associated ubiquitin ligases [54]. In view of p53 proteostasis, CHIP (C terminus of HSC70-Interacting Protein) and MDM2 E3 ubiquitin ligases display a close regulatory relationship with molecular chaperones [55]–[58]. A recent report demonstrates that in cancer cells harbouring mutant p53, HSP90 inactivates MDM2 and CHIP, thus severely impairing degradation of mutant p53. Inhibition of HSP90 by 17-Allylamino-Demethoxy Geldanamycin (17-AAG) leads to the release of mutant p53 from the complex, which enables efficient ubiquitination and degradation of p53 [58]. On the other hand, 17-AAG, by disrupting HSP90/HSF-1 association, was shown to induce a prominent heat shock transcriptional response leading to elevated levels of several stress proteins i.e. HSP27 and more importantly HSP70 [59]–[62].

In this study we provide insights into the dynamic molecular process by which mutated pro-oncogenic p53 is stabilized by HSP70. Moreover, the presence of HSP70 and MDM2 stimulate formation of aggregates containing mutant p53, MDM2 and molecular chaperones in an ATP-dependent manner. In cancer-derived cell lines, where endogenous HSP70 levels are elevated, mutant p53 protein aggregates in the nucleus with p73 inhibiting its pro-apoptotic function.

## Results

### HSP70 Contributes to Stabilization of Mutant p53 by Interfering with its Degradation

**Figure 1:**
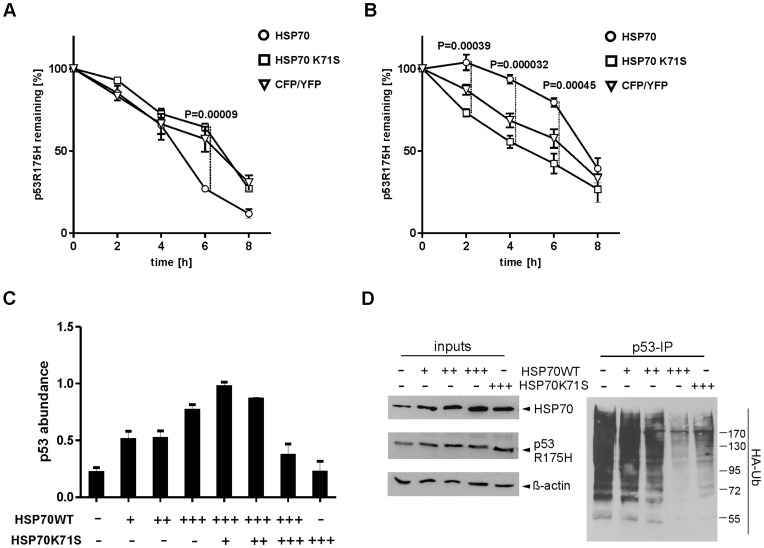
HSP70 regulates CHIP- and MDM2-dependent degradation of p53 R175H in opposite ways. Analysis of mutant p53 degradation was performed in double knockout MEF cells (*Trp53^−/−^, Mdm2^−/−^*). (**A**) CHIP-mediated degradation is accelerated by HSP70. (**B**) HSP70 WT partially inhibits MDM2-dependent p53 R175H degradation. ANOVA statistical test was carried out, where P values were counted for 3 independent experiments. (**C)** The steady-state level of p53 R175H is increased or decreased upon co-expression of WT or K71S HSP70, respectively. MEF cells were transfected with plasmids encoding p53 R175H and MDM2 together with increasing amounts of WT HSP70. Additionally, for the highest concentration of WT HSP70 titration of HSP70 K71S was applied. 24 hours later the cells were harvested and analysed by immunoblotting with p53 specific antibody (DO-1). The graph depicts the densitometric analysis performed using Image Quant software. Mean and standard deviation (s.d.) of two independent experiments are shown. (**D**) Overexpression of HSP70 downregulates the level of MDM2-dependent ubiquitination of p53 R175H**.** Cell based ubiquitination was carried out in MEF cells treated with MG132 (10 µM) for 4 hours. Mutant p53 protein was immunoprecipitated from the lysate using DO-1 antibody and analysed by western blotting using HA tag-specific antibody.

Stabilization of mutated p53 in premalignant cells can accelerate their oncogenic transformation [63], [64]. Apart from MDM2, CHIP was identified as an additional E3 ligase that plays a major role in misfolded p53 degradation [57]. In this scenario, HSP70, by apparently selective recognition of the unfolded p53 protein, promotes its CHIP–dependent ubiquitination [57], [65]. Using non-cancerous embryonic fibroblasts (MEFs) from double knockout mice (*Trp53^−/−^* and *Mdm2^−/−^*) we confirm this result (Fig. 1A). In normal or premalignant tissues of homozygous *Trp53* ^R172H^ knock-in mice, the mutated tumour suppressor is degraded mostly by MDM2. However, after cellular transformation mutant p53 becomes stabilized by unknown factor(s) [11], [12], [63], [64]. In an attempt to characterize the mechanism of cellular transformation in view of mutant p53 stabilization, we investigated the role of HSP70 in MDM2-dependent p53 R175H degradation (Fig. 1B). Contrary to CHIP-mediated degradation, MDM2-dependent degradation of p53 R175H is partially inhibited by HSP70 overexpression (Fig. 1B), while overexpression of dominant-negative HSP70 K71S mutant protein stimulates p53 R175H degradation (Fig. 1B). These results demonstrate that HSP70 plays opposing roles in CHIP- and MDM2-dependent pathways of p53 R175H degradation, accelerating the former and partially inhibiting the latter. In order to selectively analyse MDM2-dependent p53 degradation, CHIP expression was silenced by siRNA. This experiment demonstrated that the absence of CHIP does not impair HSP70-moderated, MDM2-dependent degradation of p53 R175H (Fig. S1). The effect of WT HSP70 and dominant-negative HSP70 K71S on the stability of p53 R175H is shown in Fig. 1C. WT HSP70 stabilizes mutant p53 protein in a dose dependent manner. In the presence of constant amount of WT HSP70, increasing levels of HSP70 K71S destabilize p53, most likely through increasing the rate of MDM2-dependent degradation. Interestingly, at low concentration HSP70 K71S seems to stabilize the p53 mutant, but when its level matches the level of WT HSP70, the destabilizing effect is clearly visible. In control experiments we show that HSP70 K71S indeed has a dominant-negative activity, as demonstrated by inhibition of HSP70-mediated refolding of heat-denatured luciferase (Fig. S2, SI). In order to establish whether the stabilization of p53 by increased HSP70 expression is related to p53 ubiquitination, we performed cell based ubiquitination assays. By analysing the ubiquitination profile of p53 R175H in cells expressing increasing amounts of HSP70, we can conclude that HSP70 expression levels inversely correlate with p53 ubiquitination (Fig. 1D). This effect is not due to the direct interaction of HSP70 with MDM2, as demonstrated by ELISA using purified proteins (Fig. S3, SI). Given that direct sequestration of MDM2 by HSP70 is absent, the alternative explanation of decreased p53 ubiquitination is its protection from the ubiquitin ligase by aggregation.

### HSP70 and MDM2 Enhance p53 R175H Aggregation and Alter its Subcellular Distribution

**Figure 2:**
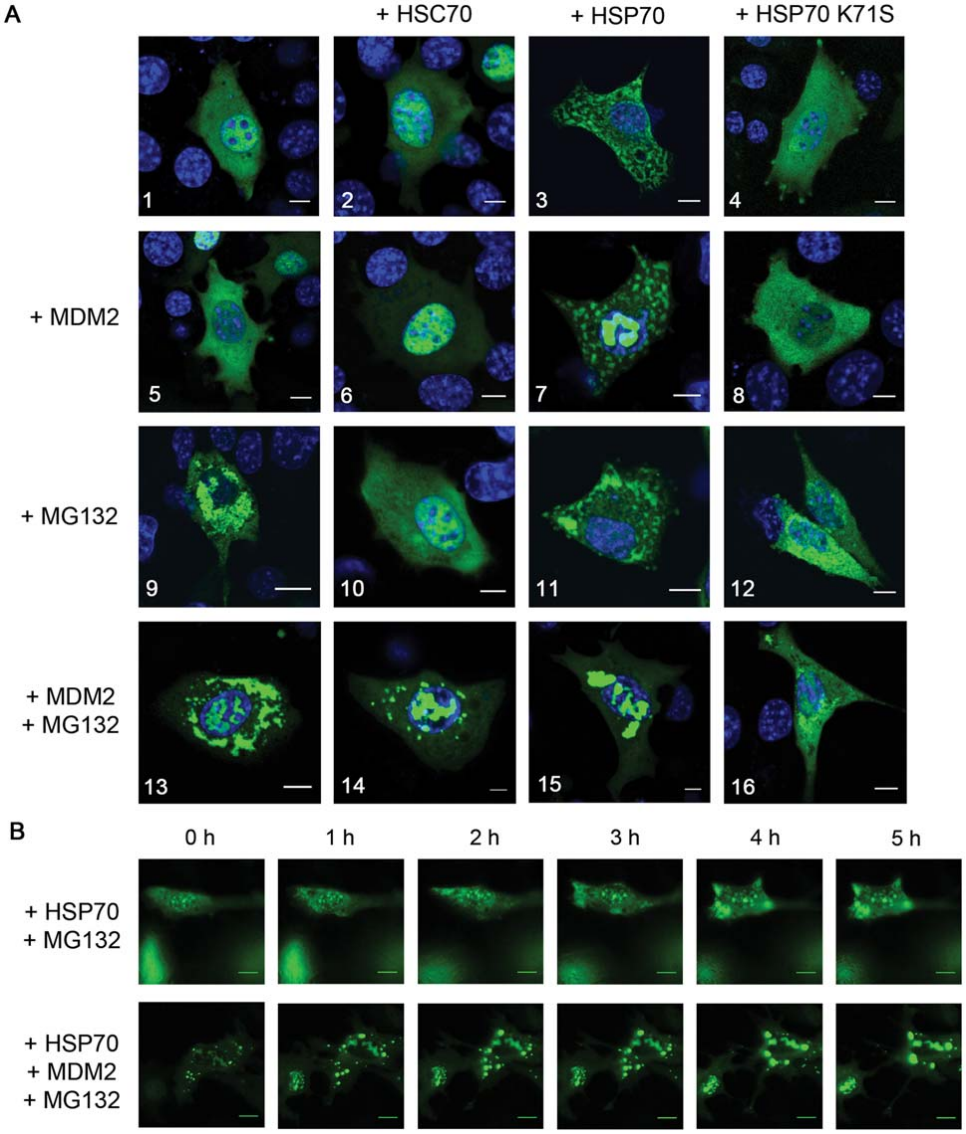
MDM2 and HSP70/HSPA family members change the localization and aggregation state of EYFP-p53 R175H in MEFs. (**A**) MEF cells (*Trp53^−/−^, Mdm2^−/−^*) were transfected with various combinations of plasmids encoding EYFP-p53 R175H, MDM2, HSC70, WT HSP70 and HSP70 K71S, as labelled above. Cells shown in 9–16 were treated with MG132 (2,5 µM) for 16 hours before imaging. Detailed description in text. Scale bar 10 µm. (**B**) MEF cells (*Trp53^−/−^, Mdm2^−/−^*) were transfected with plasmid encoding EYFP-p53 R175H together with HSP70 and optionally MDM2 (lower panel). 24 hours post-transfection cells were treated with MG132 and scanned using Olympus ScanR Station widefield fluorescent microscope (time-lapse imaging) for additional 16 hours. 0 h represents 8 hours post-transfection, subsequent frames were acquired every 1 hour. The obtained images were analysed by ScanR Analysis software.

**Figure 3:**
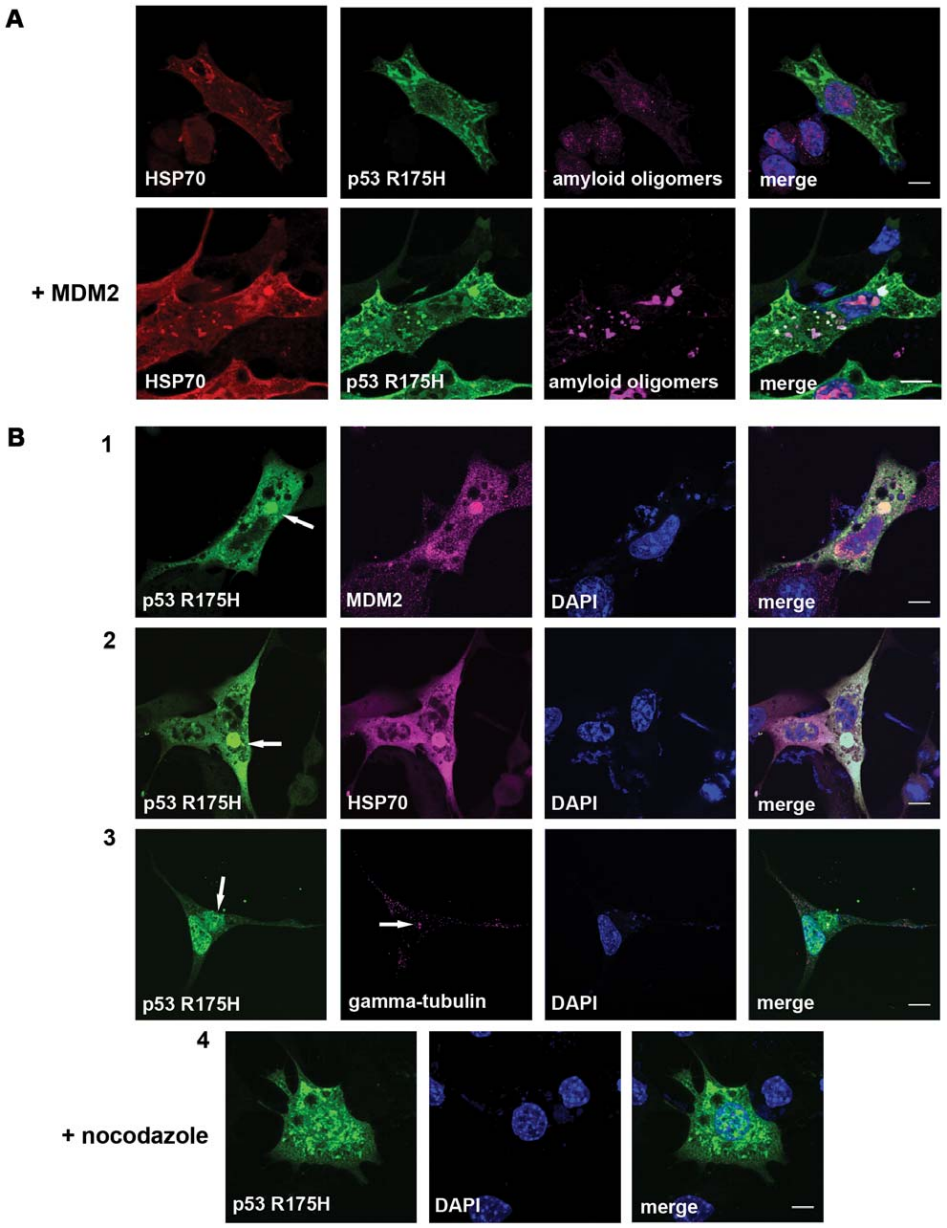
MDM2 and HSP70 colocalize with p53R175H in an aggressome. (**A**) Immunostaining of MEF cells (*Trp53^−/−^, Mdm2^−/−^*) transfected with plasmids encoding p53 R175H, HSP70 and optionally MDM2 (lower panel) shows that only in the presence of MDM2, A11 antibodies (labelling amyloid-oligomers) recognize aggregates composed of mutant p53 and HSP70. (**B**) Immunostaining of MEF cells (*Trp53^−/−^, Mdm2^−/−^*) transfected with plasmids encoding p53R175H, MDM2 and HSP70 revealed recruitment of both **(1)** the E3 ubiquitin ligase and **(2)** the chaperone to the large inclusion body. (**3)** The inclusion is formed at the centrosome marked by the presence of gamma-tubulin, suggesting that the large inclusion body containing p53 R175H is an aggresome. (**4**) Disruption of microtubules with nocodazole treatment impairs protein transport and results in the disappearance of the aggresome; smaller, scattered cytoplasmic p53-containing aggregates form instead. Scale bar 10 µm.

**Figure 4:**
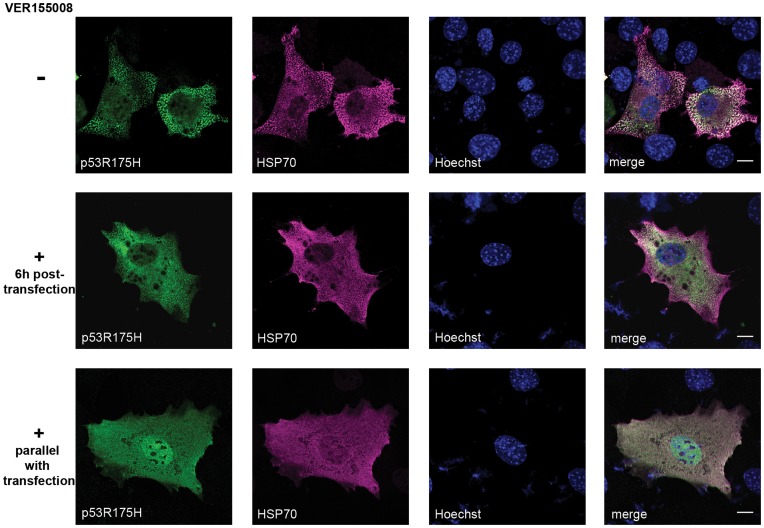
HSP70 inhibition prevents the formation of cytoplasmic p53 R175H speckles in MEFs. MEF cells (*Trp53^−/−^, Mdm2^−/−^*) were transfected with plasmids encoding p53 R175H and HSP70 and treated (or not) with HSP70 inhibitor (VER155008, Tocris Bioscience) and subsequently labelled for p53 and HSP70. In non-treated cells cytoplasmic spots containing p53 and HSP70 were observed. Small folding intermediates were visible when HSP70 inhibitor was added 6h post-transfection and no spots were detected in the case of HSP70 inhibition induced in parallel with transfection. Scale bar 10 µm.

In order to verify whether mutant p53 solubility is influenced by HSP70 we carried out confocal microscopy analysis of MEF cells (*Trp53^−/−^*, *MDM2^−/−^)* transfected with plasmids encoding EYFP-p53 R175H and optionally MDM2 in combination with different members of HSPA family of chaperones. When p53 R175H is expressed alone, diffuse nuclear and cytoplasmic localization of the protein is observed (Fig. 2A-1). Co-expression of HSC70/HSPA8 does not substantially change this distribution (Fig. 2A-2). Surprisingly, overexpression of HSP70/HSPA1-A results in p53 R175H sequestration to the cytoplasm and formation of clearly visible cytoplasmic spots (Fig. 2A-3). Under such conditions approximately 20% of transfected cells contain cytoplasmic pseudo-aggregates (Fig. S4, SI). Interestingly, MEF cells expressing p53 R273H (a mutation that unlike R175H only slightly disturbs p53 folding) also form cytoplasmic spots, but this process is much less efficient (Fig. S4, SI). Such a phenotype is not observed when the ATPase-deficient dominant-negative HSP70 K71S is overexpressed (Fig. 2A-4). Thus, we conclude that the appearance of aggregate-like structures is caused by the active HSP70 chaperoning mode, during which mutant p53 folding intermediates are formed. Such an explanation is supported by mutant p53 protein solubility tests, showing HSP70-dependent complexes to be efficiently disrupted by increasing detergent strength (Fig. S5, SI).

**Figure 5:**
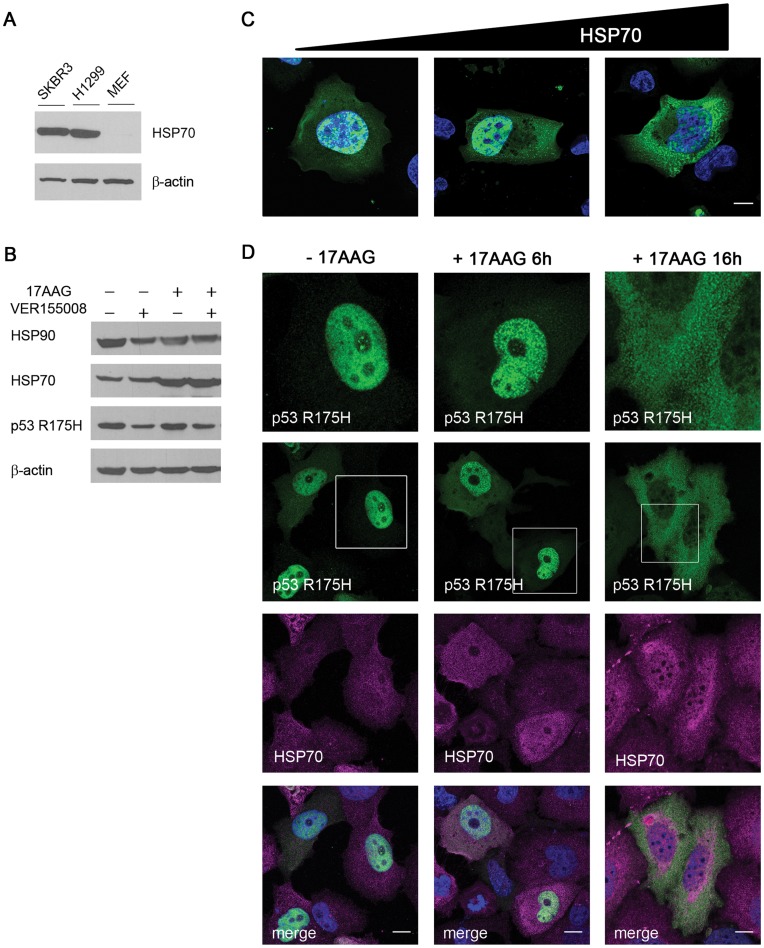
Endogenous HSP70 protein levels in H1299 cells are sufficient for nuclear aggregation of p53R175H. (**A**) SK-BR-3, H1299 and MEF cells were seeded 24 hours prior to harvesting. Immunoblotting with HSP70-specific antibody revealed high levels of HSP70 in the cancer-derived SKBR3 and H1299 cell lines when compared to double knockout MEF (*Trp53^−/−^, Mdm2^−/−^*) cells. (**B**) H1299 cells were treated (or not) with a combination of two compounds –17-AAG (1 µM, HSP90 inhibitor) and VER155008 (10 µM, HSP70 inhibitor) – for 16 hours. Immunoblotting of cell lysates shows the accumulation of HSP70 protein upon treatment with 17-AAG and the decrease in p53 R175H protein level as a result of HSP70 inhibition by VER155008. (**C**) H1299 cells were transfected with plasmids encoding p53 R175H and optionally low or high amount of HSP70 and then treated with MG132 (2 µM) for 16 hours prior to fixation. Immunostaining with p53-specific antibody revealed that the endogenous HSP70 protein level is sufficient for induction of nuclear p53 R175H aggregate formation (left panel) and that an increase in HSP70 level results in appearance of cytoplasmic p53-containing speckles (middle and right panel). (**D**) Immunofluorescence of H1299 cells treated with 17-AAG further supports the notion that endogenous HSP70 shifts the equilibrium between various oligomeric states of mutated p53. The lower level of HSP70 (6 hours after 17-AAG treatment, middle panel) triggers p53 R175H nuclear aggregation, whereas the higher level of HSP70 (16 hours after 17-AAG treatment, right panel) causes the dissociation of the nuclear aggregates into smaller speckles and their relocalization to the cytoplasm. Scale bar 10 µm.

**Figure 6:**
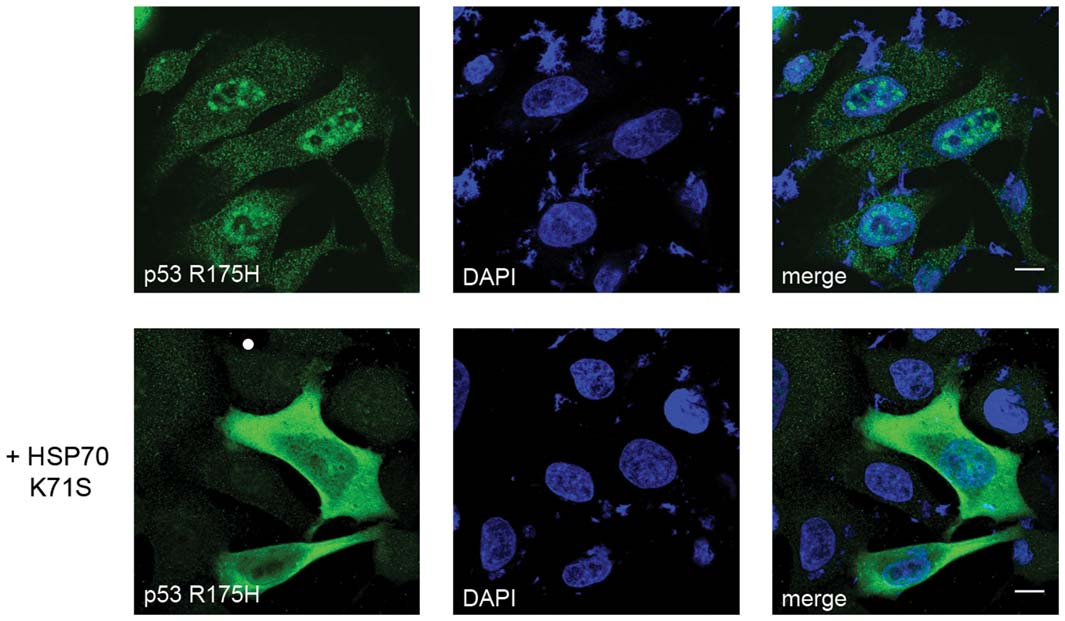
The dominant negative HSP70 K71S prevents nuclear aggregation of p53 R175H in SK-BR-3 cells. SK-BR-3 cells harbouring the R175H mutation in *TP53* gene were transfected (lower panel) with plasmid encoding HSP70 K71S and treated with MG132 (2 µM) for 16 hours. Immunofluorescent staining of p53 revealed small nuclear p53-containing aggregates in non-transfected cells (upper panel). Expression of HSP70 K71S results in cytoplasmic retention of p53 and prevents its aggregation (lower panel). Scale bar 10 µm.

**Figure 7:**
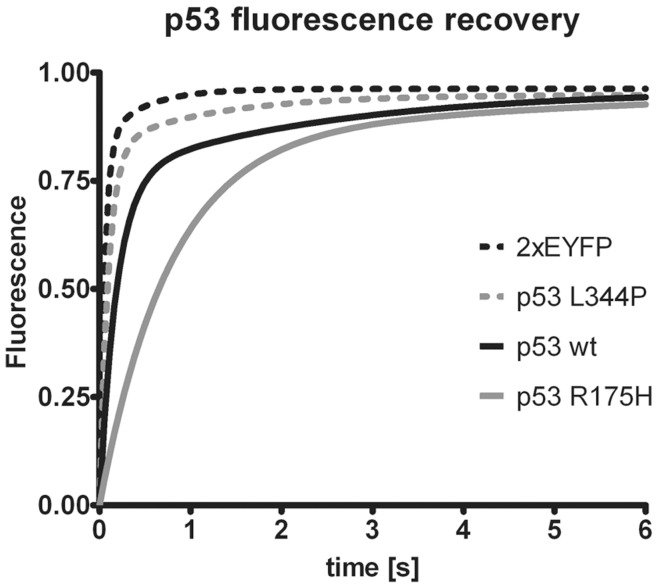
Nuclear diffusion of p53 R175H is limited. H1299 cells were transfected with plasmids encoding for indicated proteins and 24 hours post-transfection subjected to the FRAP analysis. Mobility of tandem EYFP protein and EYFP fused to indicated p53 mutant was measured in the nuclei. Recovery curves were normalized and each set of replicates was fitted to one or two phase exponential association equation with an R^2^ of no less than 0.95. The differences between all curves shown are statistically significant (larger than 95% confidence intervals, not shown for chart clarity).

**Figure 8:**
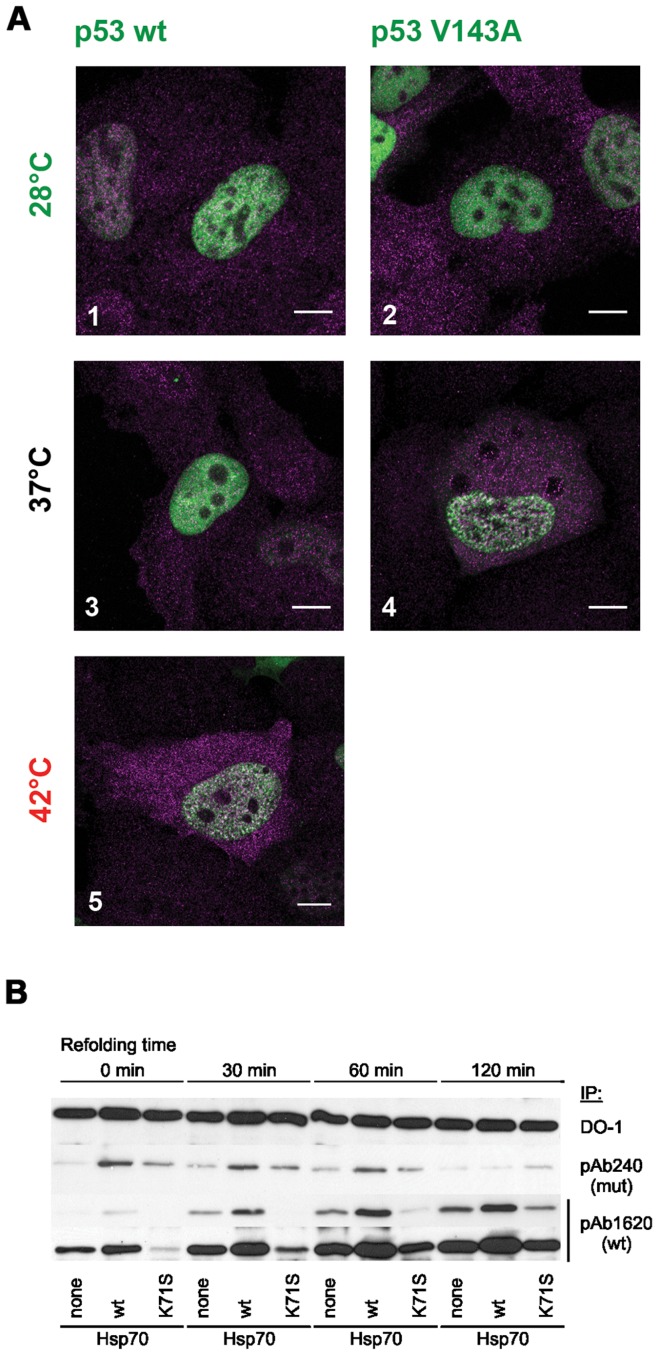
Aggregation propensity of p53 is conformation-dependent and promoted by HSP70. (**A**) Unfolding of p53 induces its nuclear aggregation**.** H1299 cells were transfected with plasmids encoding WT p53 (1, 3, 5) or p53 V143A (2, 4) and the next day incubated for 24 hours at the indicated temperature. In case of 42°C treatment, cells were incubated for 23 hours at 37°C and for 1 hour at 42°C. Small nuclear p53-containing aggregates can be seen in 4 and 5. Scale bar 10 µm. (**B**) HSP70 delays the disappearance of severely unfolded p53 temperature sensitive mutant V143A. Lysates from H1299 cells, transfected with plasmids encoding for indicated proteins, were subjected to p53 conformation-specific immunoprecipitation and analysed by western blotting. For WT conformation two exposures of the same blot are shown.

**Figure 9:**
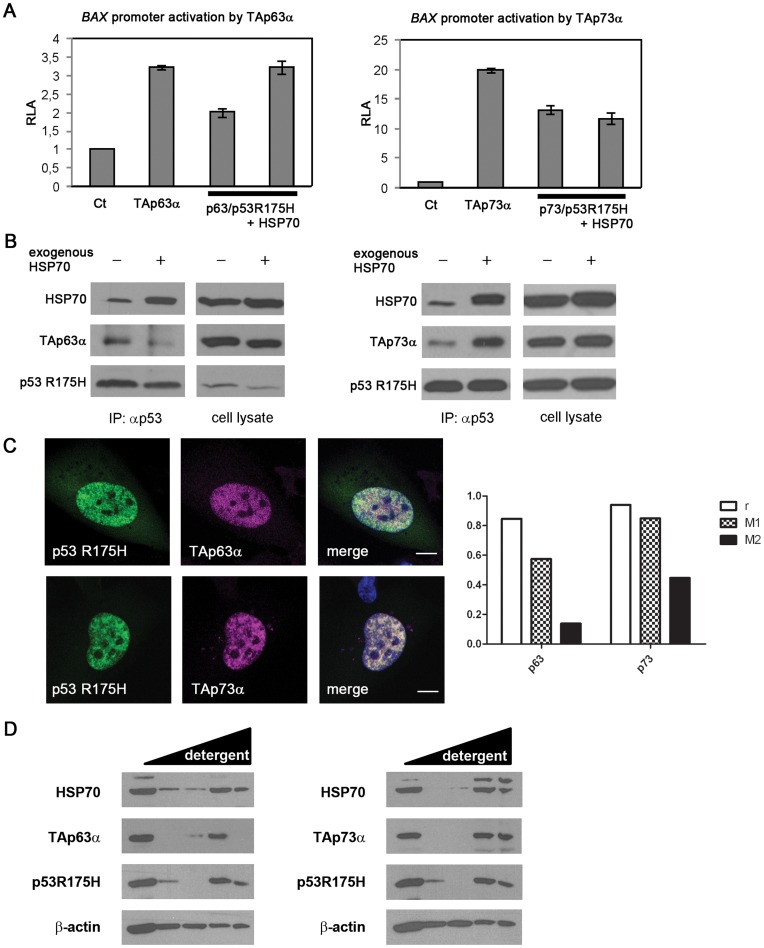
Mutant p53 sequesters p73 in cancer cells. (**A**) Overexpressed HSP70 restores transcriptional activity of TAp63α isoform inhibited by p53 R175H mutant in H1299 cells (left diagram), while TAp73α remains inhibited (right diagram). All data points were carried out in triplicate by means of Dual Luciferase Reporter Assay. (**B**) Overexpressed HSP70 molecular chaperone releases TAp63α isoform from the complex with p53 R175H mutant in H1299 cells, whereas maintains TAp73α in this interaction. 24h post-transfection cells were lysed and p53 protein was immunoprecipitated with anti-p53 antibody (DO-1). The immunoprecipitated protein complexes were subjected to Western blot analysis. (**C**) H1299 cells were transfected with plasmids encoding p53 R175H and p63 or p73 and treated with MG132 (2 µM) overnight. Labelling for specific antibodies showed that under such conditions p73 coaggregates with mutant p53 in the nucleus, while p63 remains diffused in the same compartment. Analysis performed in the ImageJ software with the JACoP plugin [88] confirmed that the colocalization is high for p73 and p53 R175H, whereas p63 overlaps with mutant p53 only partially (diagram). Two colocalization factors were determined – Pearson’s coefficient (r) and Mander’s coefficients (M1 and M2) - using threshold value of 110 for images in p63 analysis (upper panels) and 95 for p73 (lower panels). (**D**) p63 and p73 protein solubility assay performed in H1299 cells cotransfected with plasmids encoding one of those two proteins parallel with p53 R175H and HSP70 revealed that p63 is absent in the most detergent resistant fraction, while a part of p73 pool remains aggregated and is present in the Laemmli sample buffer together with mutant p53 and HSP70. Detailed description of the method available in Figures S1, S2, S3, S4, S5, S6, S7, S8, S9.

**Figure 10:**
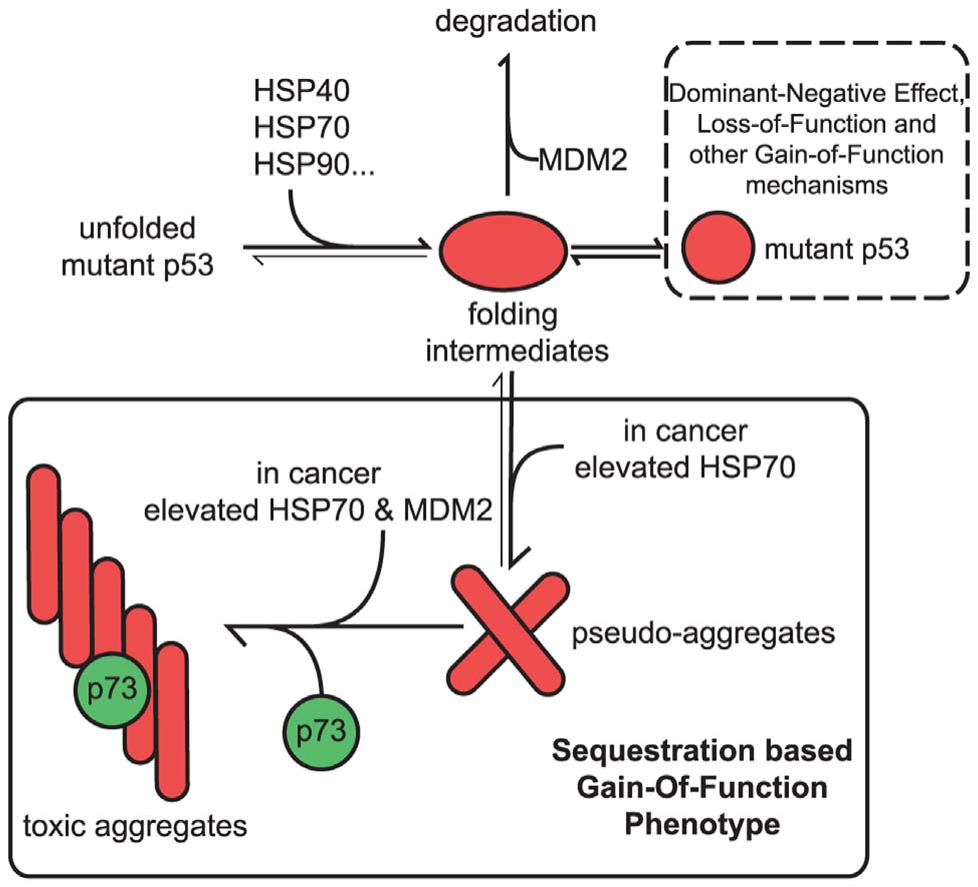
Proposed model for stabilization of mutant p53 in cancer cells. Our data suggests that the pro-oncogenic, gain-of-function phenotype of mutant p53 is governed by HSP70 and MDM2 levels. Recurrent interaction of HSP70 with the p53 polypeptide, in an ATP dependent manner, causes transient exposure of its aggregate prone domain(s). Subsequent aggregation of mutant p53 is further augmented by the MDM2-p53 allosteric interaction. This dynamic, irreversible molecular process can sequester other tumour suppressors, such as p73, thus inhibiting their activity. Pro-oncogenic activities of mutant p53 can be manifested through other, non-sequestration based mechanisms as depicted (described previously [89]). We cannot exclude the possibility that molecular chaperones, including HSP70, are also involved in these processes.

The presence of MDM2 alongside HSP70 does not significantly change p53 R175H distribution phenotype, with the only exception that a fraction of mutant p53 protein is translocated to the nucleus (Fig. 2A-7). When the cells are treated with MG132 - proteasome inhibitor leading to the accumulation of endogenous HSP70, perinuclear condensation of p53 R175H is observed (Fig. 2A-9). HSC70 and HSP70 K71S proteins efficiently inhibit this process (Fig. 2A-10, Fig.2A-12). Ectopically overexpressed HSP70 coupled with proteasome inhibition also prevents mutant p53 aggregation, additionally forming less condensed spots, which are distributed on the cell periphery (Fig. 2A-11).

The striking difference in mutant p53 localization clearly indicates that the mechanism of HSP70 activity towards mutant p53 is different from that of HSC70 or HSP70 K71S. These differences are even more pronounced when MDM2 is overexpressed alongside proteasome inhibition. Under these conditions an increased fraction of p53 is sequestered to the nucleus and structures reminiscent of nucleolar aggresomes are formed; the remaining cytoplasmic p53 also forms aggregates (Fig. 2A-13). The ATPase-dead dominant-negative HSP70 K71S is stably bound to mutant p53 thus preventing its nuclear translocation. It also seems to decrease p53 aggregation, possibly due to the passive-chaperone (holdase) activity (Fig. 2A-16). When MDM2 expression and MG132 treatment are accompanied by HSP70 overexpression, a fraction of mutant p53 becomes sequestered to nuclear aggregates but the most striking phenotype is the formation of large cytoplasmic aggregates, which in about 12% of cells merge into one large inclusion localized in the area where centrosomes are typically found (Fig. 2A-15). The formation of mutant p53 spots and their behaviour are very dynamic, as visualized by time-lapse imaging during the 16 hour period following transfection (Fig. 2B). HSP70 alone induces formation of transient p53 R175H spots. MDM2, on the other hand, not only changes the morphology of those spots (they are more compact) but also leads to merging of smaller spots into larger and finally to the formation of stable aggregates. A11 antibody stain (labelling amyloid-oligomers) further supports the hypothesis that MDM2 leads to alterations in the structure of protein complexes induced by HSP70 (Fig. 3A). The number of such dense aggregates in the cell varies from a few to one vast inclusion. Pericentrosomal localization of this inclusion strongly suggests that it represents an aggresome (Fig. 3B-3). Furthermore, treatment of the cells with nocodazole, which disrupts microtubule assembly, prevents the formation of this aggregate (Fig. 3B-4). Positive staining for both HSP70 and MDM2 confirms that these two proteins are involved in the formation of aggresomes containing mutant p53 R175H protein (Fig. 3B-1 and -2).

To additionally address the hypothesis that mutant p53 spots are folding intermediates triggered by active HSP70, we employed the HSP70 inhibitor (VER155008), which was introduced parallel or 6 hours post transfection of MEFs with plasmids encoding p53 R175H and HSP70. As shown in Fig. 4 (upper panel), overexpression of both proteins results in formation of pseudo-aggregates. However, after inhibition of HSP70 ATPase activity by the specific inhibitor, formation of these complexes was prevented (middle and lower panel in Fig. 4). We have also verified that these pseudo-aggregates apart from HSP70 contain HSP40 but do not contain HSP90 (Fig. S6, SI).

### Mutated p53 Aggregates Spontaneously in Cancer Cells

In order to further validate our findings observed in the MEF background, we employed cancer-derived cell lines that express endogenous HSP70 at high levels (Fig. 5A). Ectopic expression of p53 R175H in H1299 (p53-null) human non-small cell lung cancer (NSCLC) cells revealed distinct nuclear aggregate formation upon proteasome inhibition. Overexpression of MDM2 under those conditions led to transformation of nuclear aggregates into amyloid-like species (Fig. S7, SI). Additionally, we exploited 17-AAG, a potent HSP90 inhibitor shown to stimulate the heat shock transcription factor (HSF-1), thus further up-regulating endogenous HSP70 levels (Fig. 5B). Transient overexpression of HSP70 accompanied by proteasome inhibition as well as endogenous HSP70 increase, induced by 17-AAG treatment, resulted in the formation of cytoplasmic spots, (Fig. 5C–D) in a manner consistent with the phenotype previously observed in MEF cells.

These findings were further corroborated by analysis of p53 aggregation propensity in breast adenocarcinoma-derived SK-BR-3 cells that not only overexpress HSP70, but also harbour endogenous p53 R175H. In these cells p53 localizes predominantly in the nucleus, where upon MG132 treatment dense granules are formed (Fig. 6, upper panel). After overexpression of HSP70 K71S these aggregates disappear, showing that nuclear aggregation of endogenous p53 in SK-BR-3 cells depends on the presence of active endogenous HSP70 (Fig. 6, lower panel).

To support the notion that the speckled distribution of p53 R175H in the nucleus of H1299 and SK-BR-3 cells alike reflects aggregation, we performed nuclear Fluorescence Recovery After Photobleaching (FRAP) analysis of EYFP-p53 R175H hybrid (Fig. 7). Tandem EYFP protein, which is of the size approximately matching that of monomeric EYFP-p53 fusion protein, used as a non-aggregating and non-DNA-binding control, as expected, exhibits highest mobility. The EYFP-p53 L344P fusion protein that is strictly monomeric and does not bind DNA displays mobility similar to that of tandem EYFP. In contrast, the mobility of the EYFP-p53 WT is significantly lower than that of the control protein most likely reflecting its tetrameric state and transient DNA binding. Interestingly EYFP-p53 R175H, which lacks DNA binding potential, displays a prominent decrease in mobility (measured in areas containing no visible aggregates) when compared to EYFP-p53 WT, strongly suggesting that the diffusing p53 R175H species are in fact oligomeric and represent the early stage of aggregation.

### HSP70 Transiently Exposes the Aggregate-Prone Domain(s) of p53

The formation of p53 protein aggregates is not only limited to the p53 R175H mutation. The V143A mutation in p53 induces a conformational change upon temperature shift. At 28°C p53 V143A maintains wild-type conformation and at 37°C it adopts mutant-like conformation. In cells expressing p53 variants in their respective permissive temperatures (28°C for V143A and 28–37°C for WT) homogenous nuclear localization was observed with HSP70 localized predominantly in cytoplasm (Fig. 8A 1–3). When the cells were incubated at their respective restrictive temperatures (37°C for V143A and 42°C for WT), the formation of small nuclear aggregates that were positive for both p53 and HSP70 was observed (Fig. 8A 4–5). Together with the observation that under these conditions wild-type conformation of p53 is lost (Fig. 8B), this strongly suggests that these p53-containing aggregates form when p53 is in partially unfolded state.

Furthermore, we analyzed refolding kinetics of p53 V143A following temperature shift from 37°C to 28°C, in the presence of either WT or K71S variant of HSP70. As shown in Fig. 8B, overexpression of HSP70 increases the rate of p53 V143A refolding reaction, as visualized by increase in the amount of p53 adopting wild-type conformation that is detected by the pAb1620 antibody. After 60 min in 28°C most of p53 V143A adopts wild-type conformation. In contrast the expression of dominant-negative HSP70 K71S substantially inhibits the refolding reaction. Interestingly, expression of WT HSP70 not only accelerates the refolding reaction but also increases the fraction of p53 that adopts mutant conformation under restrictive temperature, as detected by immunoprecipitation with the pAb240 antibody directed against an internal hydrophobic p53 epitope. In addition, during refolding this misfolded form of p53 decays significantly more slowly in cells that overexpress HSP70. Such increased abundance of the p53 conformation exposing hydrophobic residues may in consequence lead to its aggregation.

It is generally accepted that the appearance of misfolded proteins induces chaperone expression. Overexpression of the p53 R175H aggregating mutant was shown to induce substantial HSP70 response, both on the transcript and protein levels [22], which we have also verified (Fig. S8, SI). This could potentially create a positive feedback loop, whereby the misfolded substrate and HSP70 reciprocally induce each other, eventually leading to aggregation.

### Mutant p53 Sequesters p73 in Cancer Cells

The p53 paralog – p73 can fulfil tumour suppressive functions when *TP53* is mutated i.e. p73 induces *BAX*-dependent apoptosis in cancer cells. However, in several types of cancer this reaction is inhibited [66]. Using H1299 background we showed that expression of TAp63 or TAp73 induces transcription from the *BAX* promoter. This transcription is inhibited by increasing concentration of mutant p53 R175H (Fig 9A) and correlates with formation of p63-p53 R175H and p73-p53 R175H complexes (Fig. 9B). Interestingly, HSP70 overproduction reactivates p63-dependent but not p73-dependent transcription from the *BAX* promoter (Fig. 9A). Under these conditions p73, but not p63, co-immunoprecipitates with p53 R175H (Fig. 9B), supporting the finding that high HSP70 levels release p63 but not p73 from the suppressive interaction with p53 R175H. We also show that, of the two paralogs, the colocalization of p73 with nuclear aggregates containing p53 R175H and HSP70 is higher (Fig. 9C). Additionally, protein solubility experiments show that p73, contrary to p63, occurs in an aggregated state, which is not disrupted by increasing detergent strength (Fig. 9D). Further increase in HSP70 level results in formation of cytoplasmic p53 R175H pseudo-aggregates, which are neither p63 nor p73 positive (Fig. S9, SI). This suggests that the cytoplasmic speckles triggered by an excess in HSP70 protein level are less toxic that nuclear aggregates containing p53 R175H, p73 and HSP70.

## Discussion

An increasing body of evidence demonstrates that the stabilization of mutant p53 is a prerequisite for its oncogenic gain-of-function activity [63]. In this study we demonstrate that overexpression of HSP70/HSPA1-A in non-cancerous mouse embryonic fibroblasts inhibits MDM2-dependent ubiquitination of p53 R175H resulting in its increased stability. This phenomenon is accompanied by coaggregation of p53 R175H with MDM2 and HSP70. These cytoplasmic aggregates occasionally form aggresomes, which are required for degradation of dysfunctional proteins through the autophagy pathway [67]. Interestingly, in the tested cancer cells, which display markedly higher levels of endogenous HSP70 than MEFs, no exogenous HSP70 is required for p53 R175H aggregation. In these cells mutant p53 aggregates spontaneously in the nucleus. Transient overexpression of WT HSP70 accompanied by proteasome inhibition, as well as endogenous HSP70 induction with 17-AAG, induces formation of cytoplasmic p53 R175H speckles, which represent folding intermediates that are soluble even in mild detergents. This effect indicates that in case of mutant p53 as a substrate, high but physiological level of HSP70 facilitates aggregation of this protein, while exceeding a certain threshold leads to dissociation of the substrate. Above this level the constant binding and release cycle limits the propensity of mutant p53 to aggregate. ATPase-dead HSP70 K71S or specific inhibitor of HSP70 ATPase, prevents formation of both nuclear p53 aggregates and cytoplasmic pseudo-aggregates, suggesting that both types of complexes are dependent on active HSP70. Strikingly, we have not observed aggresomes in cancer-derived cell lines suggesting, that in these cells degradation of proteins through autophagy is limited.

Formation of amyloid-like aggregates has been previously described for mutant p53 proteins [68] as well as for core, tetramerization and transactivation domains of wild-type p53 [69], [70]. Nevertheless, interaction with the mutant p53 protein could change the conformation of wild-type p53 and consequently lead to coaggregation of wild-type and mutant p53. Such sequestering could explain the dominant-negative activity of p53 mutants [22], [71]. A recent publication by Joost Schymkowitz laboratory, shows that aggregation of structural p53 mutants (i.e. R175H, R282W, R248Q, R249S) results from self-assembly of a conserved aggregation-nucleating sequence within the hydrophobic core of the DNA-binding domain in effect leading to a functional conversion of p53 from a tumour suppressor to an oncogene [22]. Most of those experiments, if not all, were performed on cancer cell lines, where MDM2 is present and endogenous HSP70 levels are high. Moreover, the authors present efficient heat shock response induction by structural p53 mutants. Our data not only supports the published findings, but also extends the results by providing an expanded molecular mechanism of how these cytotoxic aggregates are formed. Both HSP70 and MDM2 are required for stable mutant p53 aggregates formation. Elevated levels of MDM2 protein (expression and/or gene amplification) were observed for several types of cancer [72]–[75]. In the case of human sarcomas an aggressive subgroup was outlined, possessing both *MDM2* amplification and pro-oncogenic missense mutations within *TP53* [76]. In addition, previous studies showed that MDM2 overexpression occurs more frequently in metastatic and recurrent cancers than in primary tumours [77]. Moreover, for distant metastases MDM2 expression is a highly significant risk factor [78]. Our results indicate that MDM2 catalyzes global conformational transition of HSP70/p53 R175H pseudo-aggregate speckles to amyloid like macro-aggregates. This can be explained by the dual-site interaction mechanism that governs p53-MDM2 complex formation [79]–[81]. MDM2 has an allosteric pocket in the N-terminus that needs to be occupied to induce docking to the ubiquitination signal in the BOX-V domain of p53. Effectively this secondary docking of MDM2 to p53 distorts the overall conformation of the p53 DNA binding domain, which harbours the aggregation-nucleating sequence [22]. Thus, MDM2 by interacting with structural mutants of p53 may further enhance their aggregation propensity, by stabilizing the exposure of the aggregation-nucleating segment. Moreover, it was shown that in contrast to MDM2-mediated p53 nuclear export, p73 accumulates in the nucleus as aggregates, that colocalize with MDM2 [82]. We extend these results by showing that, at elevated HSP70 levels, p73 is stably bound to nuclear p53 R175H aggregates, while under these conditions p63 remains diffused in this compartment. These events lead to inactivation of p73 function, while p63 transcriptional activity is reactivated.

Our data indicates that HSP70 exhibits two seemingly contradictory activities: it augments p53 folding, but in case of p53 R175H conformational mutant, it can induce the formation of aggregates that contain p53 R175H, HSP70 and other components. We believe that these activities are not mutually exclusive and that the equilibrium shift towards one of these reactions is dependent on HSP70 concentration and possibly HSP70/HSP90 ratio. We noted that the HSP70-mediated aggregation reaction is ATP-dependent. This observation is consistent with a model in which HSP70 acts as an unfoldase, thus exposing substrates’ hydrophobic residues. The unfoldase activity is assigned to both HSP70 [83] and HSP90 [84]. According to those findings, molecular chaperones bind to their substrates and cause local unwinding. Subsequently, in ATP-dependent reaction, the chaperone dissociates and the substrate spontaneously refolds either to a low-affinity native state or to a high-affinity misfolded state. If the refolding is not successful, the misfolded substrate may rebind to the chaperone and undergo another unfolding cycle. The kinetics of mutant p53 refolding that we present here, indicate that HSP70 increases the initial pool of misfolded p53 and delays its disappearance before final successful refolding. In the case of p53 R175H structural mutant, the transiently unfolded protein species are likely to be more aggregation prone. In the presence of high HSP70 levels, the pool of released, transiently unfolded mutant p53 may be sufficient to initiate aggregation. These results, along with the observation that the presence of unfolded substrates induces HSP70 expression, suggest the occurrence of a self-amplifying cycle, in which unfolded p53 induces HSP70 that in turn unfolds more p53. Since protein aggregation is a concentration-dependent process, such a cycle would eventually lead to the aggregation of at least some of the p53 protein. In summary, our results suggest that HSP70 molecular chaperone binds and partially unfolds p53. Upon ATP-dependent release of HSP70 from the complex with p53, part of the unfolded p53 protein, with the help of MDM2, is captured in the aggregation-prone conformation, as depicted in the proposed model (Fig. 10).

Mouse models have recently demonstrated that the stress response induced by γ-irradiation stabilizes mutant p53 and shifts the tumour phenotype to more aggressive, as compared to treatment of *Trp53^−/−^* mice [64]. It is tempting to speculate that similar phenomena may occur in human patients who harbour mutated p53 and are treated with γ-irradiation. If that is the case, then the knowledge of patient p53 status, MDM2 protein levels may be crucial in designing the optimal therapeutic strategies. Our findings suggest a new therapeutic approach, whereby specific HSP70 inhibitors could be used synergistically with γ-irradiation in treatment of patients with mutated p53.

## Materials and Methods

### Cell Culture, Transfection and Inhibitors

MEF cells from double knockout mice (*Trp53^−/−^, MDM2^−/−^*) and SK-BR-3 (*TP53 ^R175H^*, human breast cancer cells) were maintained in DMEM supplemented with 10% FBS and antibiotics. H1299 cells (p53-null NSCLC) were cultured in RPMI 1640 medium supplemented with 10% FBS and antibiotics. Cells were grown in a 5% CO_2_ humidified incubator at 37°C (for certain experiments also at 28 or 42°C) to 70–80% confluence before transfection or other treatments. All cell lines were purchased through ATCC. MEF and SK-BR-3 transfection was performed with Attractene (Qiagen) and H1299 cells with Lipofectamine 2000 (Invitrogen) according to manufacturer’s instructions. Proteasome inhibitor MG132 (Tocris Bioscience), HSP70 inhibitor (VER155008, Tocris Bioscience), cycloheximide, nocodazole and 17-AAG (Sigma Aldrich) were used at the following concentrations 2–10 µM, 10 µM, 30 µg/ml, 100 µg/ml and 1 µM respectively.

### Plasmids

Plasmids encoding p53 and EYFP-p53 (wild-type or mutant) (Clontech, pEYFP-N1), HA tagged HSC70 and HSP70 (wild-type or mutant) were a gift from D. Walerych (pcDNA3.1 backbone). *MDM2* and *CHIP* were cloned into pcDNA3.1(+) vector (Invitrogen). The plasmid pRK5-HA-Ubiquitin WT was a gift from Ted Dawson (Addgene plasmid 17608, [85]). The plasmids encoding p63 and p73 were pRc-TAp63α and pDNA3.1-TAp73α respectively.

### Antibodies

The following antibodies for Western blotting, immunofluorescence and immunoprecipitation were used: p53 (DO-1, pAb 1620, pAb 240), p73 (rabbit polyclonal) and MDM2 (4B2) – all a kind gift from B. Vojtesek), HSP70 (SPA-812, Stressgen; 6B3, Cell Signalling), HSP40 (SPA-400, Stressgen), HSP90 (SPA-835, SPA-846, Stressgen), HA-tag (3F10, Roche Molecular Biochemicals), γ-tubulin (Abcam), p63 (4A4, Santa Cruz), amyloid oligomers (A11, Calbiochem). Secondary antibodies used in immunofluorescence were goat anti-mouse Alexa488, goat anti-rabbit Alexa555 and goat anti-rat Alexa 633 (Invitrogen).

### Analysis of Mutant p53 Degradation Rate

MEF cells were seeded in 24-well plate and transfected with plasmids encoding: p53 R175H (50ng), MDM2 or CHIP (0.5 µg) and HSP70 WT or HSP70 K71S or tandem fluorescent protein CFP/YFP - control plasmid (1 µg). After 24 h cells were treated with cycloheximide (30 µg/ml) and lysed at indicated time points. Cell lysates were analyzed by Western blotting.

### Cell Based Ubiquitination Assay

MEF cells were seeded in 6-well plate and transfected with plasmids encoding p53 R175H (0.3 µg), MDM2 (1 µg) and HA-ubiquitin (1 µg) together with plasmid encoding HSP70 WT or HSP70 K71S or CFP/YFP - control plasmid (2 µg). Subsequent steps of the assay were carried out as previously described [86].

### Immunoprecipitation

H1299 cells were seeded in 6-well plates and transfected with plasmids encoding: TAp63α (4 µg), TAp73α (0.25 µg), p53 R175H (1 µg - Co-IP with TAp63α, 0.25 µg - Co-IP with TAp73α), and HA-HSP70 (2 µg). Subsequent steps of the assay were carried out as previously described [84].

### Conformation-specific Immunoprecipitation

H1299 cells were transfected with p53 V143A and optionally, with WT HSP70 or dominant-negative HSP70 K71S. 24 hours post-transfection cells were moved from 37°C to 28°C and lysed at times indicated. p53 from lysates was immunoprecipitated using conformation-specific antibodies and analyzed by Western blotting. Detailed description of the method in [48].

### Dual Luciferase Reporter Assay

H1299 cells were seeded in 24-well plates and transfected with plasmids encoding: Renilla luciferase reporter (pCMV-RL –30 ng), Firefly luciferase reporter gene under *BAX* promoter, (*BAX*-luc –70 ng), TAp63α (0.3 µg), TAp73α (50 ng), p53 R175H (0.5 µg), HA-HSP70 (0.75 µg). Subsequent steps of the assay were carried out as previously described [84], [87].

### Immunofluorescence

To ensure correct morphology the cells were seeded at low density on collagen-coated sterile coverslips. Prior to staining cells were fixed with 4% paraformaldehyde for 10 min at room temperature, permeabilized with 1% Triton X-100 in PBS and then blocked with 2% BSA and 0.1% Triton X-100 in PBS for 10 minutes at RT. All primary antibodies were diluted 1∶200 in blocking buffer and incubated with the coverslips for 1 hour. All secondary antibodies were diluted 1∶500 in the same buffer and incubated with the coverslips for 30 minutes. Following DAPI/Hoechst staining (1∶5000) and washing, the coverslips were mounted with Gel Mount (Sigma). Images were acquired with a Zeiss LSM5 Exciter confocal microscope or Olympus ScanR Station fluorescent microscope for statistical analysis.

### Live Cell Imaging

Cells grown in 8-well Chambered Coverglass LabTek were transfected with plasmids encoding proteins indicated in figure legends. Optionally, cells were treated overnight with MG132 and/or nocodazole or DMSO as a control. 24 hours post-transfection cells were stained with Hoechst and imaged using confocal microscope Zeiss LSM710 or Olympus ScanR Station wide field fluorescent microscope (time-lapse imaging).

### Fluorescence Recovery After Photobleaching

Images were acquired with Leica DM IRE/TCS SP2 confocal microscope equipped with full environment control. Imaging was performed at 32×256 pixels, 5% 514 nm laser power and bleaching (circle of 2 µm diameter) with one pass at 100% power of both 488 and 514 nm laser lines. Beam expander was set to 1 and pinhole diameter to 3 AU. 5 pre-bleaching frames, 1 bleach frame and 60 post-bleaching frames were acquired in bidirectional “fly” mode. At least 10 cells for each experimental variant were imaged. Fluorescence intensity in the bleached region was corrected for total specimen bleaching.

## Supporting Information

Figure S1 **MDM2-dependent degradation of p53 R175H is partially inhibited by HSP70 upon CHIP silencing.** Double knockout MEF cells (*Trp53* ^−/−^, *Mdm2* ^−/−^) were transfected with siRNA (Ambion) against CHIP and (**A**) 24 hours later with plasmids encoding p53 R175H together with MDM2 and optionally with HSP70 WT, HSP70 K71S or a control plasmid. 24 hours post-transfection cells were treated with 30 µg/ml of cycloheximide (CHX) and collected every two hours. The level of p53 protein was detected using western blotting and DO-1 antibody. Curves were plotted based on densitometry. (**B**) The efficacy of siRNA (10 nM) against CHIP is shown.
(TIF)

Figure S2 **HSP70 K71S acts in a dominant-negative manner towards WT HSP70 in a Luciferase Refolding Assay.** Reactivation of denatured firefly luciferase was performed essentially as described (Walerych et al, 2004) with minor modifications. Briefly, luciferase (Promega) at a concentration of 2.5 mM was denatured for 5 min at 50°C in a stability buffer containing 40 mM Tris-HCl pH 7.4, 50 mM KCl, 8 mM MgSO_4,_ 10% glycerol, 1% BSA, 0.25% Triton X-100 and 0.5 µM HSP90. After cooling down the denatured luciferase mix was diluted 15-fold with a renaturation buffer: 40 mM Tris pH 7.4, 15 mM MgCl_2_, 50 mM KCl, 5 mM DTT, 20 mM CP, 0.02 u/µl CK, 1 mM ATP, 5 µM HSP70, 1.0 µM Hdj1/DNAJB1 and increasing concentration (from 5 to 15 µM) of HSP70 K71S. Renaturation was carried out at room temperature. At time points ranging from 0 to 120 min, 5 µl aliquots were taken, and the activity of renatured luciferase was measured in a luminometer (BMG Labtechnologies) using Bright-Glo substrate (Promega).
(TIF)

Figure S3 ***In vitro*** **interactions of MDM2 with molecular chaperones and p53 by ELISA technique.** Approximately 400 ng of purified p53 (as a positive control) or the appropriate molecular chaperones, or BSA (as a negative control) were coated onto 96-well ELISA plate in the Coating Buffer (25 mM HEPES, pH 7.4, 100 mM KCl). Following the 1 h coating at 25°C, blocking procedure was continued for additional 1 h with Washing Buffer (25 mM HEPES, pH 7.4, 100 mM KCl, 2 mg/ml BSA). After removing of unbound proteins, increasing amounts of MDM2 in the Reaction Buffer (25 mM HEPES, pH 7.4, 100 mM KCl, 10 mM MgCl_2_, 2 mM DTT, 5% glycerol, 0.05% Triton X-100, 2 mg/ml BSA) were added. The reaction proceeded for 45 min and the ELISA plate was washed three times with the Washing Buffer. Subsequently primary antibodies against MDM2 (H-221 - Santa Cruz, or 4B2– kind gift from Borek Vojtesek) were added and incubated at 25°C for 2 h. Then the plate was washed three times and the appropriate secondary antibodies conjugated to horseradish peroxidase (Bio-Rad) were added for 1 h. The plate was then washed three times and the TMB peroxidase EIA substrate kit (Bio-Rad) was employed to visualize HRP. The absorbance was read at 450 nm in the microplate reader (Bio-Rad). Interactions between MDM2 and three molecular chaperones are comparable to BSA, which is a negative control.
(TIF)

Figure S4 **HSP70 induces pseudo-aggregates of both structural and DNA-contact p53 mutants.** Statistical analysis of MEF cells containing mutant p53 cytoplasmic speckles was performed using Olympus ScanR Station widefield fluorescent microscope. MEF cells were transfected with plasmids encoding p53 R175H or p53 R273H together with HSP70 in three repeats for each combination. 48 hours post-transfection cells were fixed and stained for p53. 528 cells were analyzed in case of the structural p53 R175H mutant and 781 cells for the contact p53 R273H variant. P value was counted based on t test. Mean and s.d. of three independent experiments are shown. In MEF cells transfected only with plasmids encoding p53 R175H or p53 R273H but not HSP70 no cytoplasmic spots were observed.
(TIF)

Figure S5 **Mutant p53 protein solubility.** Protein solubility assay in buffers with increasing detergent strength reveals that HSP70 facilitates p53 R175H protein diffusion. MEF cells were transfected with plasmids encoding proteins indicated in figure legends and 48 hours later collected and snap frozen. Subsequently cells were resuspended in ice-cold 25 mM HEPES pH 7.5 buffer (supplemented with protease inhibitor cocktail - Roche), incubated for 15 minutes and centrifuged prior to supernatant collection. This procedure was repeated twice with the buffer supplemented with detergents (0,2% NP-40 and 0,1% Triton X-100, respectively). Afterwards the pellet was resuspended in modified RIPA buffer (50 mM Tris-HCl pH 7.4, 150 mM NaCl, 0.5 mM EDTA, 1% Triton X-100, 0.1% SDS, 0.5% Deoxycholate) and finally in the Laemmli sample buffer (60 mM Tris-HCl pH 6.8, 10% Glycerol, 2% SDS, 5% 2-Mercaptoethanol). Total protein normalization was followed by Western Blot analysis.
(TIF)

Figure S6 **Composition of cytoplasmic p53 R175H protein spots.** MEF cells were transfected with plasmids encoding p53 R175H and HSP70. 48 hours post-transfection the cells were fixed and labeled for mutant p53, HSP70, HSP40 and HSP90. Nucleus was stained with Hoechst (blue). Scale bar 10 µm.
(TIF)

Figure S7 **MDM2 induces amyloid-like aggregation of p53 R175H in the nucleus of H1299 cells.** H1299 cells were transfected with plasmid encoding p53 R175H and optionally MDM2. 6 hours post-transfection medium was changed for one containing MG132 proteasome inhibitor (2 µM). 16 h later the cells were fixed and labeled for mutant p53 and amyloid oligomers. Nucleus was stained with Hoechst (blue). Scale bar 10 µm.
(TIF)

Figure S8 **The level of HSP70 in MEFs increases upon overexpression of** ***TP53*** **R175H.** MEF cells were transfected with increasing amount of plasmid encoding p53 R175H. 24 hours post-transfection the cells were lysed and analyzed by western blotting.
(TIF)

Figure S9 **p53 R175H cytoplasmic pseudo-aggregates do not sequester transcription factors.** H1299 cells were transfected with plasmids encoding p53 R175H and HSP70 parallel with either p63 or p73. 6 hours post-transfection medium was changed for one containing MG132 proteasome inhibitor (2 µM). 16 h later the cells were fixed and labeled for mutant p53, HSP70, p63 and p73. Nucleus was stained with Hoechst (blue). Scale bar 10 µm.
(TIF)

## References

[1] Oren M (2003) Decision making by p53: life, death and cancer. Cell Death Differ 10: 431–442. 12719720 10.1038/sj.cdd.4401183

[2] Wu X, Bayle JH, Olson D, Levine AJ (1993) The p53-mdm-2 autoregulatory feedback loop. Genes Dev 7: 1126–1132. 8319905 10.1101/gad.7.7a.1126

[3] Haupt Y, Maya R, Kazaz A, Oren M (1997) Mdm2 promotes the rapid degradation of p53. Nature 387: 296–299. 9153395 10.1038/387296a0

[4] Honda R, Tanaka H, Yasuda H (1997) Oncoprotein MDM2 is a ubiquitin ligase E3 for tumor suppressor p53. FEBS Lett 420: 25–27. 9450543 10.1016/s0014-5793(97)01480-4

[5] Kubbutat MH, Jones SN, Vousden KH (1997) Regulation of p53 stability by Mdm2. Nature 387: 299–303. 9153396 10.1038/387299a0

[6] Gajjar M, Candeias MM, Malbert-Colas L, Mazars A, Fujita J, et al (2012) The p53 mRNA-Mdm2 interaction controls Mdm2 nuclear trafficking and is required for p53 activation following DNA damage. Cancer Cell 21: 25–35. 22264786 10.1016/j.ccr.2011.11.016

[7] Vogelstein B, Lane D, Levine AJ (2000) Surfing the p53 network. Nature 408: 307–310. 11099028 10.1038/35042675

[8] Bartek J, Bartkova J, Vojtesek B, Staskova Z, Lukas J, et al (1991) Aberrant expression of the p53 oncoprotein is a common feature of a wide spectrum of human malignancies. Oncogene 6: 1699–1703. 1923535

[9] Hollstein M, Sidransky D, Vogelstein B, Harris CC (1991) p53 mutations in human cancers. Science 253: 49–53. 1905840 10.1126/science.1905840

[10] Muller PA, Caswell PT, Doyle B, Iwanicki MP, Tan EH, et al (2009) Mutant p53 drives invasion by promoting integrin recycling. Cell 139: 1327–1341. 20064378 10.1016/j.cell.2009.11.026

[11] Lang GA, Iwakuma T, Suh YA, Liu G, Rao VA, et al (2004) Gain of function of a p53 hot spot mutation in a mouse model of Li-Fraumeni syndrome. Cell 119: 861–872. 15607981 10.1016/j.cell.2004.11.006

[12] Olive KP, Tuveson DA, Ruhe ZC, Yin B, Willis NA, et al (2004) Mutant p53 gain of function in two mouse models of Li-Fraumeni syndrome. Cell 119: 847–860. 15607980 10.1016/j.cell.2004.11.004

[13] Blandino G, Levine AJ, Oren M (1999) Mutant p53 gain of function: differential effects of different p53 mutants on resistance of cultured cells to chemotherapy. Oncogene 18: 477–485. 9927204 10.1038/sj.onc.1202314

[14] Zalcenstein A, Stambolsky P, Weisz L, Muller M, Wallach D, et al (2003) Mutant p53 gain of function: repression of CD95(Fas/APO-1) gene expression by tumor-associated p53 mutants. Oncogene 22: 5667–5676. 12944915 10.1038/sj.onc.1206724

[15] Weisz L, Zalcenstein A, Stambolsky P, Cohen Y, Goldfinger N, et al (2004) Transactivation of the EGR1 gene contributes to mutant p53 gain of function. Cancer Res 64: 8318–8327. 15548700 10.1158/0008-5472.CAN-04-1145

[16] Strano S, Dell’Orso S, Di Agostino S, Fontemaggi G, Sacchi A, et al (2007) Mutant p53: an oncogenic transcription factor. Oncogene 26: 2212–2219. 17401430 10.1038/sj.onc.1210296

[17] Maslon MM, Hupp TR (2010) Drug discovery and mutant p53. Trends Cell Biol 20: 542–555. 20656489 10.1016/j.tcb.2010.06.005

[18] Muller PA, Vousden KH, Norman JC (2011) p53 and its mutants in tumor cell migration and invasion. J Cell Biol 192: 209–218. 21263025 10.1083/jcb.201009059 PMC3172183

[19] Strano S, Munarriz E, Rossi M, Cristofanelli B, Shaul Y, et al (2000) Physical and functional interaction between p53 mutants and different isoforms of p73. J Biol Chem 275: 29503–29512. 10884390 10.1074/jbc.M003360200

[20] Gaiddon C, Lokshin M, Ahn J, Zhang T, Prives C (2001) A subset of tumor-derived mutant forms of p53 down-regulate p63 and p73 through a direct interaction with the p53 core domain. Mol Cell Biol 21: 1874–1887. 11238924 10.1128/MCB.21.5.1874-1887.2001 PMC86759

[21] Li Y, Prives C (2007) Are interactions with p63 and p73 involved in mutant p53 gain of oncogenic function? Oncogene 26: 2220–2225. 17401431 10.1038/sj.onc.1210311

[22] Xu J, Reumers J, Couceiro JR, De Smet F, Gallardo R, et al (2011) Gain of function of mutant p53 by coaggregation with multiple tumor suppressors. Nat Chem Biol 7: 285–295. 21445056 10.1038/nchembio.546

[23] Petitjean A, Mathe E, Kato S, Ishioka C, Tavtigian SV, et al (2007) Impact of mutant p53 functional properties on TP53 mutation patterns and tumor phenotype: lessons from recent developments in the IARC TP53 database. Hum Mutat 28: 622–629. 17311302 10.1002/humu.20495

[24] Acin S, Li Z, Mejia O, Roop DR, El-Naggar AK, et al (2011) Gain-of-function mutant p53 but not p53 deletion promotes head and neck cancer progression in response to oncogenic K-ras. J Pathol 225: 479–489. 21952947 10.1002/path.2971 PMC4302750

[25] Trbusek M, Smardova J, Malcikova J, Sebejova L, Dobes P, et al (2011) Missense mutations located in structural p53 DNA-binding motifs are associated with extremely poor survival in chronic lymphocytic leukemia. J Clin Oncol 29: 2703–2708. 21606432 10.1200/JCO.2011.34.7872

[26] Lo Nigro C, Vivenza D, Monteverde M, Lattanzio L, Gojis O, et al (2012) High frequency of complex TP53 mutations in CNS metastases from breast cancer. Br J Cancer 106: 397–404. 22187033 10.1038/bjc.2011.464 PMC3261685

[27] Dai C, Whitesell L, Rogers AB, Lindquist S (2007) Heat shock factor 1 is a powerful multifaceted modifier of carcinogenesis. Cell 130: 1005–1018. 17889646 10.1016/j.cell.2007.07.020 PMC2586609

[28] King FW, Wawrzynow A, Hohfeld J, Zylicz M (2001) Co-chaperones Bag-1, Hop and Hsp40 regulate Hsc70 and Hsp90 interactions with wild-type or mutant p53. Embo J 20: 6297–6305. 11707401 10.1093/emboj/20.22.6297 PMC125724

[29] Zylicz M, King FW, Wawrzynow A (2001) Hsp70 interactions with the p53 tumour suppressor protein. Embo J 20: 4634–4638. 11532927 10.1093/emboj/20.17.4634 PMC125613

[30] Calderwood SK, Khaleque MA, Sawyer DB, Ciocca DR (2006) Heat shock proteins in cancer: chaperones of tumorigenesis. Trends Biochem Sci 31: 164–172. 16483782 10.1016/j.tibs.2006.01.006

[31] Garrido C, Brunet M, Didelot C, Zermati Y, Schmitt E, et al (2006) Heat shock proteins 27 and 70: anti-apoptotic proteins with tumorigenic properties. Cell Cycle 5: 2592–2601. 17106261 10.4161/cc.5.22.3448

[32] Steiner K, Graf M, Hecht K, Reif S, Rossbacher L, et al (2006) High HSP70-membrane expression on leukemic cells from patients with acute myeloid leukemia is associated with a worse prognosis. Leukemia 20: 2076–2079. 16990768 10.1038/sj.leu.2404391

[33] Tsutsumi S, Beebe K, Neckers L (2009) Impact of heat-shock protein 90 on cancer metastasis. Future Oncol 5: 679–688. 19519207 10.2217/fon.09.30 PMC2714685

[34] Volloch VZ, Sherman MY (1999) Oncogenic potential of Hsp72. Oncogene 18: 3648–3651. 10380887 10.1038/sj.onc.1202525

[35] Rohde M, Daugaard M, Jensen MH, Helin K, Nylandsted J, et al (2005) Members of the heat-shock protein 70 family promote cancer cell growth by distinct mechanisms. Genes Dev 19: 570–582. 15741319 10.1101/gad.305405 PMC551577

[36] Langer T, Lu C, Echols H, Flanagan J, Hayer MK, et al (1992) Successive action of DnaK, DnaJ and GroEL along the pathway of chaperone-mediated protein folding. Nature 356: 683–689. 1349157 10.1038/356683a0

[37] Hartl FU, Bracher A, Hayer-Hartl M (2011) Molecular chaperones in protein folding and proteostasis. Nature 475: 324–332. 21776078 10.1038/nature10317

[38] Jiang J, Ballinger CA, Wu Y, Dai Q, Cyr DM, et al (2001) CHIP is a U-box-dependent E3 ubiquitin ligase: identification of Hsc70 as a target for ubiquitylation. J Biol Chem 276: 42938–42944. 11557750 10.1074/jbc.M101968200

[39] Hohfeld J, Cyr DM, Patterson C (2001) From the cradle to the grave: molecular chaperones that may choose between folding and degradation. EMBO Rep 2: 885–890. 11600451 10.1093/embo-reports/kve206 PMC1084084

[40] Esser C, Alberti S, Hohfeld J (2004) Cooperation of molecular chaperones with the ubiquitin/proteasome system. Biochim Biophys Acta 1695: 171–188. 15571814 10.1016/j.bbamcr.2004.09.020

[41] Kriegenburg F, Ellgaard L, Hartmann-Petersen R (2012) Molecular chaperones in targeting misfolded proteins for ubiquitin-dependent degradation. Febs J 279: 532–542. 22177318 10.1111/j.1742-4658.2011.08456.x

[42] Skowyra D, Georgopoulos C, Zylicz M (1990) The E. coli dnaK gene product, the hsp70 homolog, can reactivate heat-inactivated RNA polymerase in an ATP hydrolysis-dependent manner. Cell 62: 939–944. 2203539 10.1016/0092-8674(90)90268-j

[43] Ziemienowicz A, Skowyra D, Zeilstra-Ryalls J, Fayet O, Georgopoulos C, et al (1993) Both the Escherichia coli chaperone systems, GroEL/GroES and DnaK/DnaJ/GrpE, can reactivate heat-treated RNA polymerase. Different mechanisms for the same activity. J Biol Chem 268: 25425–25431. 7902351

[44] Parsell DA, Kowal AS, Singer MA, Lindquist S (1994) Protein disaggregation mediated by heat-shock protein Hsp104. Nature 372: 475–478. 7984243 10.1038/372475a0

[45] Zietkiewicz S, Krzewska J, Liberek K (2004) Successive and synergistic action of the Hsp70 and Hsp100 chaperones in protein disaggregation. J Biol Chem 279: 44376–44383. 15302880 10.1074/jbc.M402405200

[46] Zietkiewicz S, Lewandowska A, Stocki P, Liberek K (2006) Hsp70 chaperone machine remodels protein aggregates at the initial step of Hsp70-Hsp100-dependent disaggregation. J Biol Chem 281: 7022–7029. 16415353 10.1074/jbc.M507893200

[47] Nollen EA, Salomons FA, Brunsting JF, van der Want JJ, Sibon OC, et al (2001) Dynamic changes in the localization of thermally unfolded nuclear proteins associated with chaperone-dependent protection. Proc Natl Acad Sci U S A 98: 12038–12043. 11572931 10.1073/pnas.201112398 PMC59763

[48] Walerych D, Olszewski MB, Gutkowska M, Helwak A, Zylicz M, et al (2009) Hsp70 molecular chaperones are required to support p53 tumor suppressor activity under stress conditions. Oncogene 28: 4284–4294. 19749793 10.1038/onc.2009.281

[49] Ravagnan L, Gurbuxani S, Susin SA, Maisse C, Daugas E, et al (2001) Heat-shock protein 70 antagonizes apoptosis-inducing factor. Nat Cell Biol 3: 839–843. 11533664 10.1038/ncb0901-839

[50] Zylicz M, LeBowitz JH, McMacken R, Georgopoulos C (1983) The dnaK protein of Escherichia coli possesses an ATPase and autophosphorylating activity and is essential in an in vitro DNA replication system. Proc Natl Acad Sci U S A 80: 6431–6435. 6314326 10.1073/pnas.80.21.6431 PMC390127

[51] Liberek K, Marszalek J, Ang D, Georgopoulos C, Zylicz M (1991) Escherichia coli DnaJ and GrpE heat shock proteins jointly stimulate ATPase activity of DnaK. Proc Natl Acad Sci U S A 88: 2874–2878. 1826368 10.1073/pnas.88.7.2874 PMC51342

[52] Liberek K, Skowyra D, Zylicz M, Johnson C, Georgopoulos C (1991) The Escherichia coli DnaK chaperone, the 70-kDa heat shock protein eukaryotic equivalent, changes conformation upon ATP hydrolysis, thus triggering its dissociation from a bound target protein. J Biol Chem 266: 14491–14496. 1830586

[53] Pelham HR (1986) Speculations on the functions of the major heat shock and glucose-regulated proteins. Cell 46: 959–961. 2944601 10.1016/0092-8674(86)90693-8

[54] Dreiseidler M, Dick N, Hohfeld J (2012) Analysis of chaperone-assisted ubiquitylation. Methods Mol Biol 832: 473–487. 22350907 10.1007/978-1-61779-474-2_34

[55] Peng Y, Chen L, Li C, Lu W, Chen J (2001) Inhibition of MDM2 by hsp90 contributes to mutant p53 stabilization. J Biol Chem 276: 40583–40590. 11507088 10.1074/jbc.M102817200

[56] Esser C, Scheffner M, Hohfeld J (2005) The chaperone associated ubiquitin ligase CHIP is able to target p53 for proteasomal degradation. J Biol Chem. 10.1074/jbc.M501574200 15911628

[57] Muller P, Hrstka R, Coomber D, Lane DP, Vojtesek B (2008) Chaperone-dependent stabilization and degradation of p53 mutants. Oncogene 27: 3371–3383. 18223694 10.1038/sj.onc.1211010

[58] Li D, Marchenko ND, Schulz R, Fischer V, Velasco-Hernandez T, et al (2011) Functional inactivation of endogenous MDM2 and CHIP by HSP90 causes aberrant stabilization of mutant p53 in human cancer cells. Mol Cancer Res 9: 577–588. 21478269 10.1158/1541-7786.MCR-10-0534 PMC3097033

[59] Bagatell R, Paine-Murrieta GD, Taylor CW, Pulcini EJ, Akinaga S, et al (2000) Induction of a heat shock factor 1-dependent stress response alters the cytotoxic activity of hsp90-binding agents. Clin Cancer Res 6: 3312–3318. 10955818

[60] Winklhofer KF, Reintjes A, Hoener MC, Voellmy R, Tatzelt J (2001) Geldanamycin restores a defective heat shock response in vivo. J Biol Chem 276: 45160–45167. 11574536 10.1074/jbc.M104873200

[61] Guo F, Rocha K, Bali P, Pranpat M, Fiskus W, et al (2005) Abrogation of heat shock protein 70 induction as a strategy to increase antileukemia activity of heat shock protein 90 inhibitor 17-allylamino-demethoxy geldanamycin. Cancer Res 65: 10536–10544. 16288046 10.1158/0008-5472.CAN-05-1799

[62] McCollum AK, Teneyck CJ, Sauer BM, Toft DO, Erlichman C (2006) Up-regulation of heat shock protein 27 induces resistance to 17-allylamino-demethoxygeldanamycin through a glutathione-mediated mechanism. Cancer Res 66: 10967–10975. 17108135 10.1158/0008-5472.CAN-06-1629

[63] Terzian T, Suh YA, Iwakuma T, Post SM, Neumann M, et al (2008) The inherent instability of mutant p53 is alleviated by Mdm2 or p16INK4a loss. Genes Dev 22: 1337–1344. 18483220 10.1101/gad.1662908 PMC2377188

[64] Suh YA, Post SM, Elizondo-Fraire AC, Maccio DR, Jackson JG, et al (2011) Multiple stress signals activate mutant p53 in vivo. Cancer Res 71: 7168–7175. 21983037 10.1158/0008-5472.CAN-11-0459 PMC3320147

[65] Esser C, Scheffner M, Hohfeld J (2005) The chaperone-associated ubiquitin ligase CHIP is able to target p53 for proteasomal degradation. J Biol Chem 280: 27443–27448. 15911628 10.1074/jbc.M501574200

[66] Coates PJ (2006) Regulating p73 isoforms in human tumours. J Pathol 210: 385–389. 17044134 10.1002/path.2080

[67] Fortun J, Dunn WA Jr, Joy S, Li J, Notterpek L (2003) Emerging role for autophagy in the removal of aggresomes in Schwann cells. J Neurosci 23: 10672–10680. 14627652 10.1523/JNEUROSCI.23-33-10672.2003 PMC6740927

[68] Levy CB, Stumbo AC, Ano Bom AP, Portari EA, Cordeiro Y, et al (2011) Co-localization of mutant p53 and amyloid-like protein aggregates in breast tumors. Int J Biochem Cell Biol 43: 60–64. 21056685 10.1016/j.biocel.2010.10.017

[69] Bom AP, Freitas MS, Moreira FS, Ferraz D, Sanches D, et al (2010) The p53 core domain is a molten globule at low pH: functional implications of a partially unfolded structure. J Biol Chem 285: 2857–2866. 19933157 10.1074/jbc.M109.075861 PMC2807339

[70] Silva JL, Vieira TC, Gomes MP, Bom AP, Lima LM, et al (2010) Ligand binding and hydration in protein misfolding: insights from studies of prion and p53 tumor suppressor proteins. Acc Chem Res 43: 271–279. 19817406 10.1021/ar900179t PMC2825094

[71] Milner J, Medcalf EA (1991) Cotranslation of activated mutant p53 with wild type drives the wild-type p53 protein into the mutant conformation. Cell 65: 765–774. 2040013 10.1016/0092-8674(91)90384-b

[72] Momand J, Jung D, Wilczynski S, Niland J (1998) The MDM2 gene amplification database. Nucleic Acids Res 26: 3453–3459. 9671804 10.1093/nar/26.15.3453 PMC147746

[73] Bond GL, Hirshfield KM, Kirchhoff T, Alexe G, Bond EE, et al (2006) MDM2 SNP309 accelerates tumor formation in a gender-specific and hormone-dependent manner. Cancer Res 66: 5104–5110. 16707433 10.1158/0008-5472.CAN-06-0180

[74] Sheikh MS, Shao ZM, Hussain A, Fontana JA (1993) The p53-binding protein MDM2 gene is differentially expressed in human breast carcinoma. Cancer Res 53: 3226–3228. 8324731

[75] Brekman A, Singh KE, Polotskaia A, Kundu N, Bargonetti J (2011) A p53-independent role of Mdm2 in estrogen-mediated activation of breast cancer cell proliferation. Breast Cancer Res 13: R3. 21223569 10.1186/bcr2804 PMC3109566

[76] Cordon-Cardo C, Latres E, Drobnjak M, Oliva MR, Pollack D, et al (1994) Molecular abnormalities of mdm2 and p53 genes in adult soft tissue sarcomas. Cancer Res 54: 794–799. 8306343

[77] Ladanyi M, Cha C, Lewis R, Jhanwar SC, Huvos AG, et al (1993) MDM2 gene amplification in metastatic osteosarcoma. Cancer Res 53: 16–18. 8416741

[78] Mathew R, Arora S, Khanna R, Mathur M, Shukla NK, et al (2002) Alterations in p53 and pRb pathways and their prognostic significance in oesophageal cancer. Eur J Cancer 38: 832–841. 11937319 10.1016/s0959-8049(02)00007-2

[79] Shimizu H, Burch LR, Smith AJ, Dornan D, Wallace M, et al (2002) The conformationally flexible S9–S10 linker region in the core domain of p53 contains a novel MDM2 binding site whose mutation increases ubiquitination of p53 in vivo. J Biol Chem 277: 28446–28458. 11925449 10.1074/jbc.M202296200

[80] Ma J, Martin JD, Zhang H, Auger KR, Ho TF, et al (2006) A second p53 binding site in the central domain of Mdm2 is essential for p53 ubiquitination. Biochemistry 45: 9238–9245. 16866370 10.1021/bi060661u

[81] Wallace M, Worrall E, Pettersson S, Hupp TR, Ball KL (2006) Dual-site regulation of MDM2 E3-ubiquitin ligase activity. Mol Cell 23: 251–263. 16857591 10.1016/j.molcel.2006.05.029

[82] Gu J, Nie L, Wiederschain D, Yuan ZM (2001) Identification of p53 sequence elements that are required for MDM2-mediated nuclear export. Mol Cell Biol 21: 8533–8546. 11713288 10.1128/MCB.21.24.8533-8546.2001 PMC100016

[83] Sharma SK, De los Rios P, Christen P, Lustig A, Goloubinoff P (2010) The kinetic parameters and energy cost of the Hsp70 chaperone as a polypeptide unfoldase. Nat Chem Biol 6: 914–920. 20953191 10.1038/nchembio.455

[84] Walerych D, Gutkowska M, Klejman MP, Wawrzynow B, Tracz Z, et al (2010) ATP binding to Hsp90 is sufficient for effective chaperoning of p53 protein. J Biol Chem 285: 32020–32028. 20688913 10.1074/jbc.M110.112110 PMC2952203

[85] Lim KL, Chew KC, Tan JM, Wang C, Chung KK, et al (2005) Parkin mediates nonclassical, proteasomal-independent ubiquitination of synphilin-1: implications for Lewy body formation. J Neurosci 25: 2002–2009. 15728840 10.1523/JNEUROSCI.4474-04.2005 PMC6726069

[86] Zhu Q, Yao J, Wani G, Wani MA, Wani AA (2001) Mdm2 mutant defective in binding p300 promotes ubiquitination but not degradation of p53: evidence for the role of p300 in integrating ubiquitination and proteolysis. J Biol Chem 276: 29695–29701. 11340074 10.1074/jbc.M102634200

[87] Wawrzynow B, Pettersson S, Zylicz A, Bramham J, Worrall E, et al (2009) A function for the RING finger domain in the allosteric control of MDM2 conformation and activity. J Biol Chem 284: 11517–11530. 19188367 10.1074/jbc.M809294200 PMC2670157

[88] Bolte S, Cordelieres FP (2006) A guided tour into subcellular colocalization analysis in light microscopy. J Microsc 224: 213–232. 17210054 10.1111/j.1365-2818.2006.01706.x

[89] Neilsen PM, Noll JE, Suetani RJ, Schulz RB, Al-Ejeh F, et al (2011) Mutant p53 uses p63 as a molecular chaperone to alter gene expression and induce a pro-invasive secretome. Oncotarget 2: 1203–1217. 22203497 10.18632/oncotarget.382 PMC3282078

