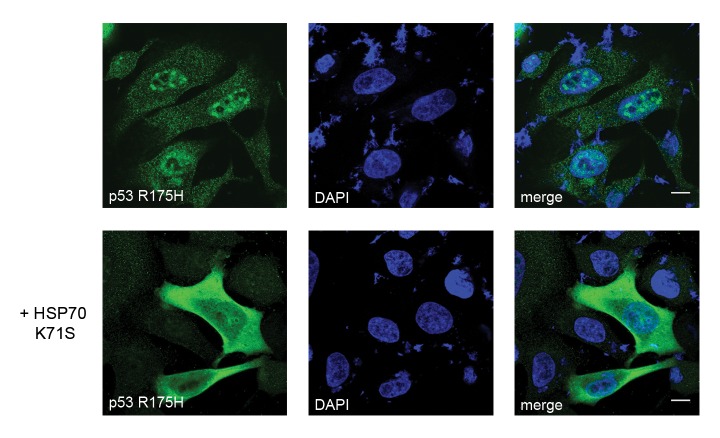# Correction: Molecular Mechanism of Mutant p53 Stabilization: The Role of HSP70 and MDM2

**DOI:** 10.1371/annotation/5f4c812a-79d4-44eb-9890-6a8de45c303b

**Published:** 2013-08-12

**Authors:** Milena Wiech, Maciej B. Olszewski, Zuzanna Tracz-Gaszewska, Bartosz Wawrzynow, Maciej Zylicz, Alicja Zylicz

There is a white circle in Figure 6 that was erroneously introduced into a panel on the left side. The following link provides the correct version of Figure 6: 

**Figure:**