# Correction: Molecular Mechanism of Mutant p53 Stabilization: The Role of HSP70 and MDM2

**DOI:** 10.1371/journal.pone.0361672

**Published:** 2026-10-29

**Authors:** Milena Wiech, Maciej B. Olszewski, Zuzanna Tracz-Gaszewska, Bartosz Wawrzynow, Maciej Zylicz, Alicja Zylicz

Following the publication of this article [1,2], concerns were raised regarding Figs 1, 2, and S5. Specifically,

- In the Fig 1D p53-IP panel, there appears to be a vertical discontinuity between lanes 3 and 4.
- The Fig 2B + HSP70 + MG132 4 h panel appears more similar to the Fig 2B + HSP70 + MG132 5 h panel than expected.
- The Fig 2B + HSP70 + MDM2 + MG132 2 h panel appears more similar to the Fig 2B + HSP70 + MDM2 + MG132 3 h panel than expected.
- In Fig S5, the p53 R175H panel appears similar to the β-actin panel.

Corresponding author MZ stated that the + HSP70 + MG132 4 h, + HSP70 + MDM2 + MG132 3 h, and + HSP70 + MDM2 + MG132 0 h panels in Fig 2B are incorrect. They also stated that the timestamps in Fig 2B in [1] are incorrect, and should instead be 0–3.75 h at 45 min intervals. The corresponding author stated that the underlying images for Fig 2B are no longer available but provided videos stated to correspond to the same data as in Fig 2B demonstrating the time dependent formation of p53R175H pseudo aggregates in the presence of HSP70 overproduction and amyloid-like aggregates in the presence of HSP70 & MDM2 overproduction (S1 File). Corresponding author MZ also provided an updated version of Fig 2B (S4 File) with corrected timestamps based on the video in S1 File. A member of the *PLOS One* Editorial Board stated that S4 File is supported by the videos in S1 File.

Regarding Fig S5, corresponding author MZ stated that the β-actin panel is incorrect. A correct Fig S5 has been provided here as S2 File where the β-actin panel has been replaced with the correct panel from the original experiments. Corresponding author MZ stated that the underlying blots for Fig S5 are no longer available.

Regarding Fig 1, corresponding author MZ stated that the samples were developed on the same gel, that the vertical discontinuity between lanes 3 and 4 is the result of an artifact created during film development, and that the image is not modified. They also stated that the underlying blots for Fig 1D are no longer available.

The available underlying images for Fig 2A are provided here in S3 File. Corresponding author MZ stated that the remainder of the underlying data for [1] are no longer available, with the exception of the data underlying Figs 3, 6, 9B, S2, S3, S6, and S7, which are available upon request from authors MW and AZ.

The *PLOS One* Editors have concerns related to aspects of the peer review process. Readers are encouraged to interpret the article [1] with consideration of this issue.

## Supporting information

S1 File Videos assembled from images in support of Fig 2B, some of which are included in Fig 2B in [1]. Time has been added for illustration purposes to indicate the time span of the cell scan.
(ZIP)

S2 File Updated version of Figure S5. (ZIP)

S3 File Underlying images in support of the following Fig 2A panels: + MDM2 + MG132 + HSC70, + MDM2 + HSP70 K71S, Blank + HSP70 K71S, + MDM2 Blank, Blank Blank, + MG132 + HSP70, + MG132 Blank, and + MG132 + HSP70 K71S. (ZIP)

S4 File An updated version of Fig 2B based on the video in S1 File. MEF cells (*Trp53*^*-/-*^, *Mdm2*^*-/-*^) were transfected with a plasmid encoding EYFP-p53 R175H, together with HSP70 and optionally MDM2 (lower panel), and treated with MG132 24 hours post-transfection. Time-lapse imaging was performed using an Olympus ScanR Station widefield fluorescence microscope for at least 16 hours, with frames acquired every 45 minutes/0.75h. An approximately 4-hour scanning interval corresponding to the highest dynamics of changes was selected for the figure. “0 h” represents 8 hours after the addition of MG132. “0 h” corresponds to 11.25 h in the video for the (+HSP70) condition, and to 6.75 h for the (HSP70 + MDM2) condition. The resulting images were analyzed using ScanR Analysis software. Scale bar: 50 µm.
(PNG)
