# Supplementary figures and images for: Molecular Mechanism of Mutant p53 Stabilization: The Role of HSP70 and MDM2
Source: PLoS One. 2012 Dec 12;7(12):e51426. doi: 10.1371/journal.pone.0051426 (PMC3520893; doi:10.1371/journal.pone.0051426)

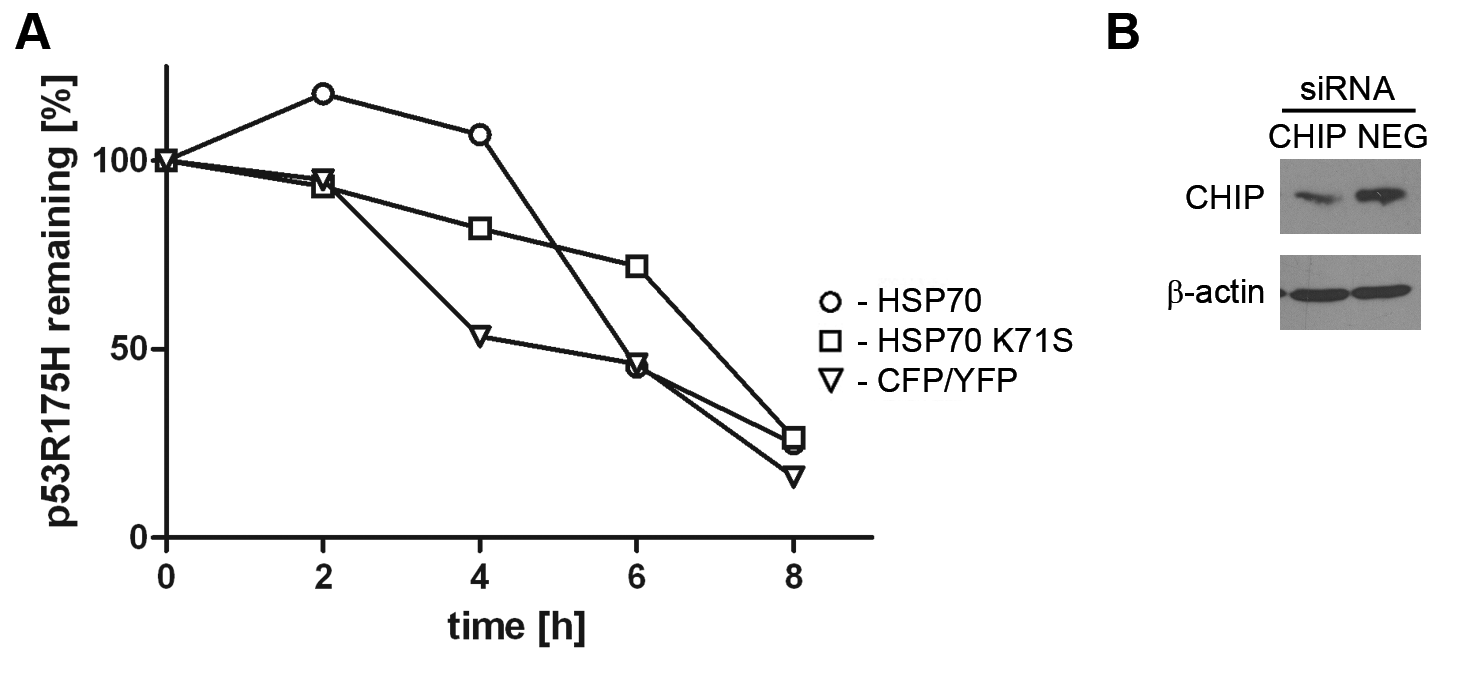

Supplement: Figure S1 — MDM2-dependent degradation of p53 R175H is partially inhibited by HSP70 upon CHIP silencing. Double knockout MEF cells (Trp53 −/−, Mdm2 −/−) were transfected with siRNA (Ambion) against CHIP and (A) 24 hours later with plasmids encoding p53 R175H together with MDM2 and optionally with HSP70 WT, HSP70 K71S or a control plasmid. 24 hours post-transfection cells were treated with 30 µg/ml of cycloheximide (CHX) and collected every two hours. The level of p53 protein was detected using western blotting and DO-1 antibody. Curves were plotted based on densitometry. (B) The efficacy of siRNA (10 nM) against CHIP is shown. (TIF) [file pone.0051426.s001.tif]

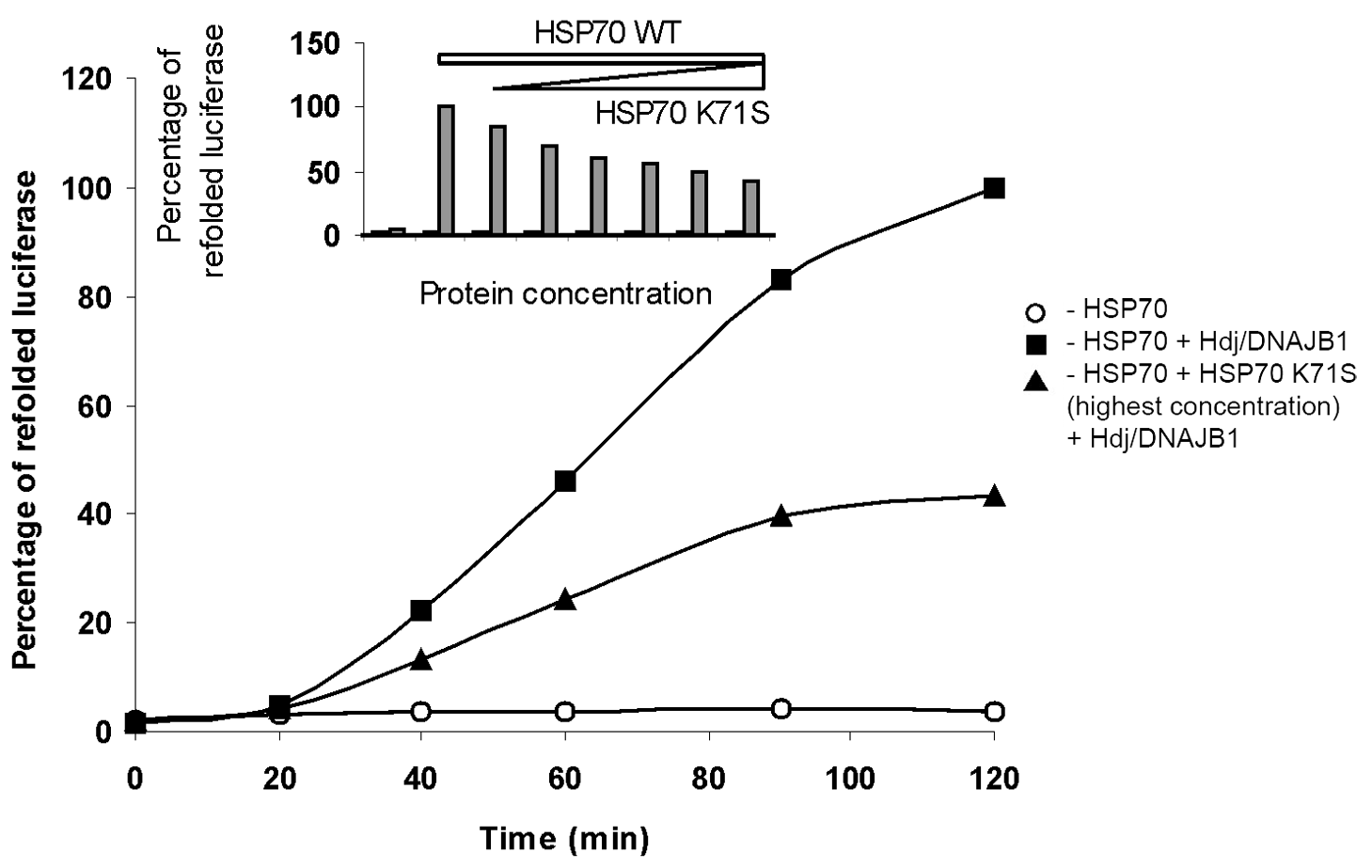

Supplement: Figure S2 — HSP70 K71S acts in a dominant-negative manner towards WT HSP70 in a Luciferase Refolding Assay. Reactivation of denatured firefly luciferase was performed essentially as described (Walerych et al, 2004) with minor modifications. Briefly, luciferase (Promega) at a concentration of 2.5 mM was denatured for 5 min at 50°C in a stability buffer containing 40 mM Tris-HCl pH 7.4, 50 mM KCl, 8 mM MgSO4, 10% glycerol, 1% BSA, 0.25% Triton X-100 and 0.5 µM HSP90. After cooling down the denatured luciferase mix was diluted 15-fold with a renaturation buffer: 40 mM Tris pH 7.4, 15 mM MgCl2, 50 mM KCl, 5 mM DTT, 20 mM CP, 0.02 u/µl CK, 1 mM ATP, 5 µM HSP70, 1.0 µM Hdj1/DNAJB1 and increasing concentration (from 5 to 15 µM) of HSP70 K71S. Renaturation was carried out at room temperature. At time points ranging from 0 to 120 min, 5 µl aliquots were taken, and the activity of renatured luciferase was measured in a luminometer (BMG Labtechnologies) using Bright-Glo substrate (Promega). (TIF) [file pone.0051426.s002.tif]

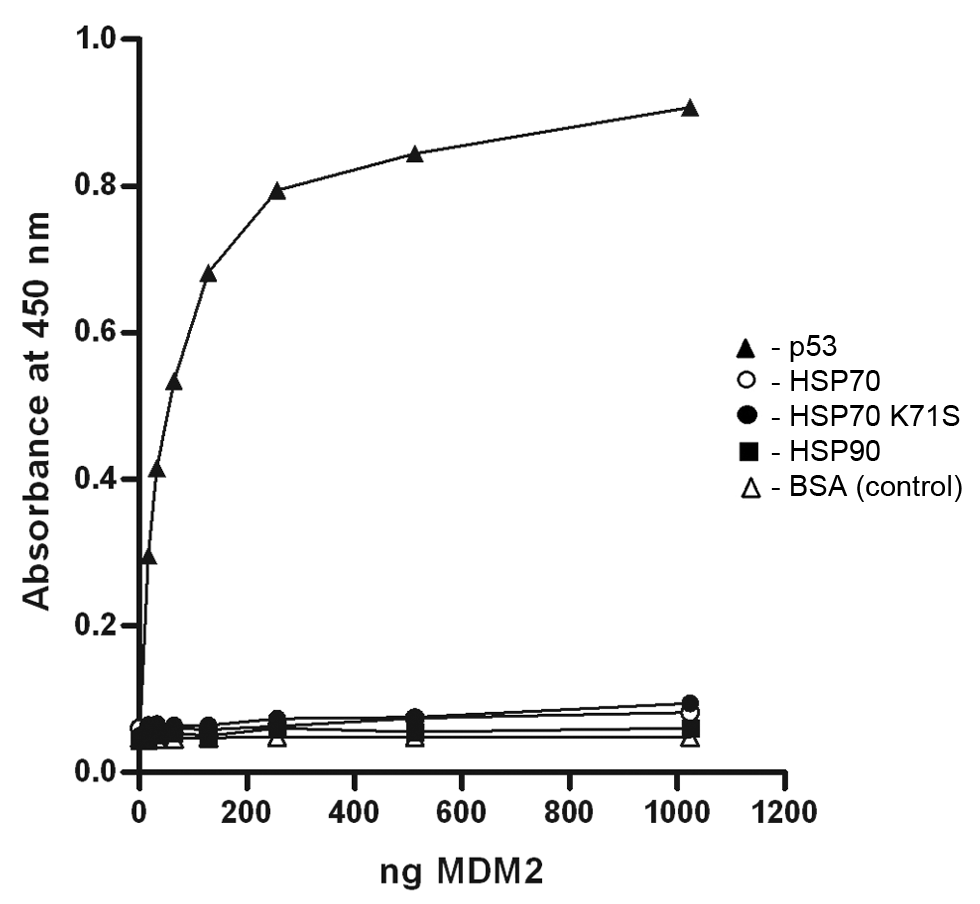

Supplement: Figure S3 — In vitro interactions of MDM2 with molecular chaperones and p53 by ELISA technique. Approximately 400 ng of purified p53 (as a positive control) or the appropriate molecular chaperones, or BSA (as a negative control) were coated onto 96-well ELISA plate in the Coating Buffer (25 mM HEPES, pH 7.4, 100 mM KCl). Following the 1 h coating at 25°C, blocking procedure was continued for additional 1 h with Washing Buffer (25 mM HEPES, pH 7.4, 100 mM KCl, 2 mg/ml BSA). After removing of unbound proteins, increasing amounts of MDM2 in the Reaction Buffer (25 mM HEPES, pH 7.4, 100 mM KCl, 10 mM MgCl2, 2 mM DTT, 5% glycerol, 0.05% Triton X-100, 2 mg/ml BSA) were added. The reaction proceeded for 45 min and the ELISA plate was washed three times with the Washing Buffer. Subsequently primary antibodies against MDM2 (H-221 - Santa Cruz, or 4B2– kind gift from Borek Vojtesek) were added and incubated at 25°C for 2 h. Then the plate was washed three times and the appropriate secondary antibodies conjugated to horseradish peroxidase (Bio-Rad) were added for 1 h. The plate was then washed three times and the TMB peroxidase EIA substrate kit (Bio-Rad) was employed to visualize HRP. The absorbance was read at 450 nm in the microplate reader (Bio-Rad). Interactions between MDM2 and three molecular chaperones are comparable to BSA, which is a negative control. (TIF) [file pone.0051426.s003.tif]

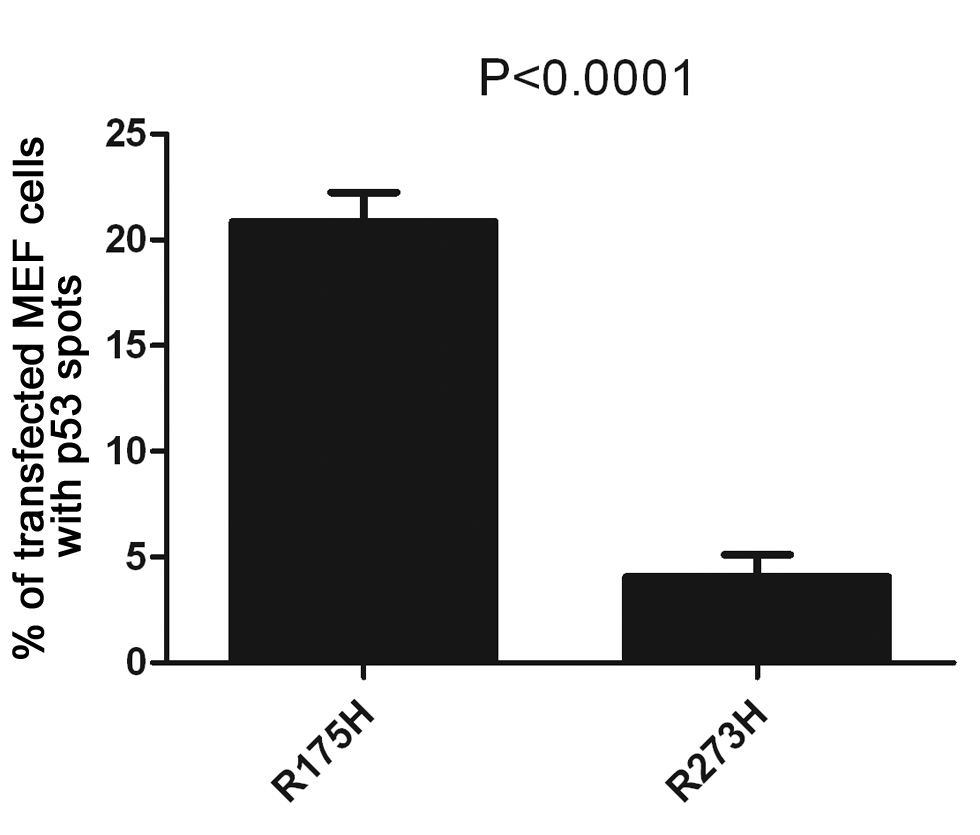

Supplement: Figure S4 — HSP70 induces pseudo-aggregates of both structural and DNA-contact p53 mutants. Statistical analysis of MEF cells containing mutant p53 cytoplasmic speckles was performed using Olympus ScanR Station widefield fluorescent microscope. MEF cells were transfected with plasmids encoding p53 R175H or p53 R273H together with HSP70 in three repeats for each combination. 48 hours post-transfection cells were fixed and stained for p53. 528 cells were analyzed in case of the structural p53 R175H mutant and 781 cells for the contact p53 R273H variant. P value was counted based on t test. Mean and s.d. of three independent experiments are shown. In MEF cells transfected only with plasmids encoding p53 R175H or p53 R273H but not HSP70 no cytoplasmic spots were observed. (TIF) [file pone.0051426.s004.tif]

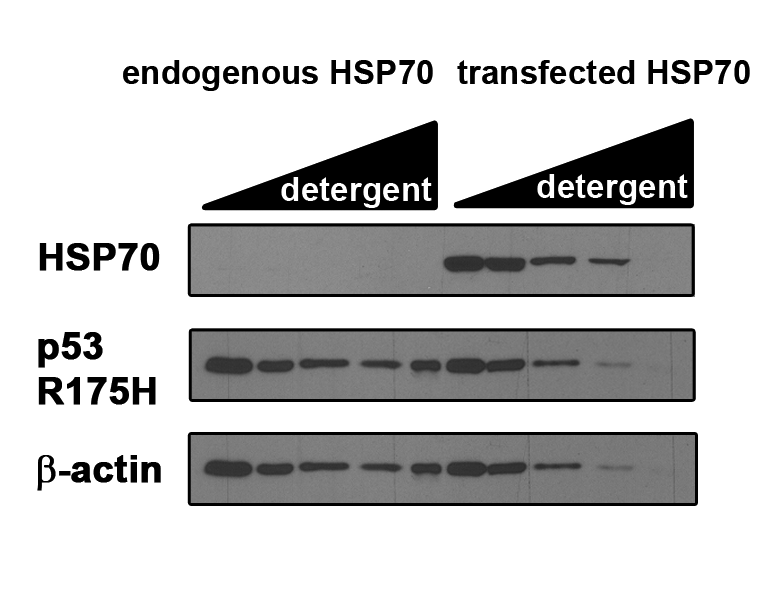

Supplement: Figure S5 — Mutant p53 protein solubility. Protein solubility assay in buffers with increasing detergent strength reveals that HSP70 facilitates p53 R175H protein diffusion. MEF cells were transfected with plasmids encoding proteins indicated in figure legends and 48 hours later collected and snap frozen. Subsequently cells were resuspended in ice-cold 25 mM HEPES pH 7.5 buffer (supplemented with protease inhibitor cocktail - Roche), incubated for 15 minutes and centrifuged prior to supernatant collection. This procedure was repeated twice with the buffer supplemented with detergents (0,2% NP-40 and 0,1% Triton X-100, respectively). Afterwards the pellet was resuspended in modified RIPA buffer (50 mM Tris-HCl pH 7.4, 150 mM NaCl, 0.5 mM EDTA, 1% Triton X-100, 0.1% SDS, 0.5% Deoxycholate) and finally in the Laemmli sample buffer (60 mM Tris-HCl pH 6.8, 10% Glycerol, 2% SDS, 5% 2-Mercaptoethanol). Total protein normalization was followed by Western Blot analysis. (TIF) [file pone.0051426.s005.tif]

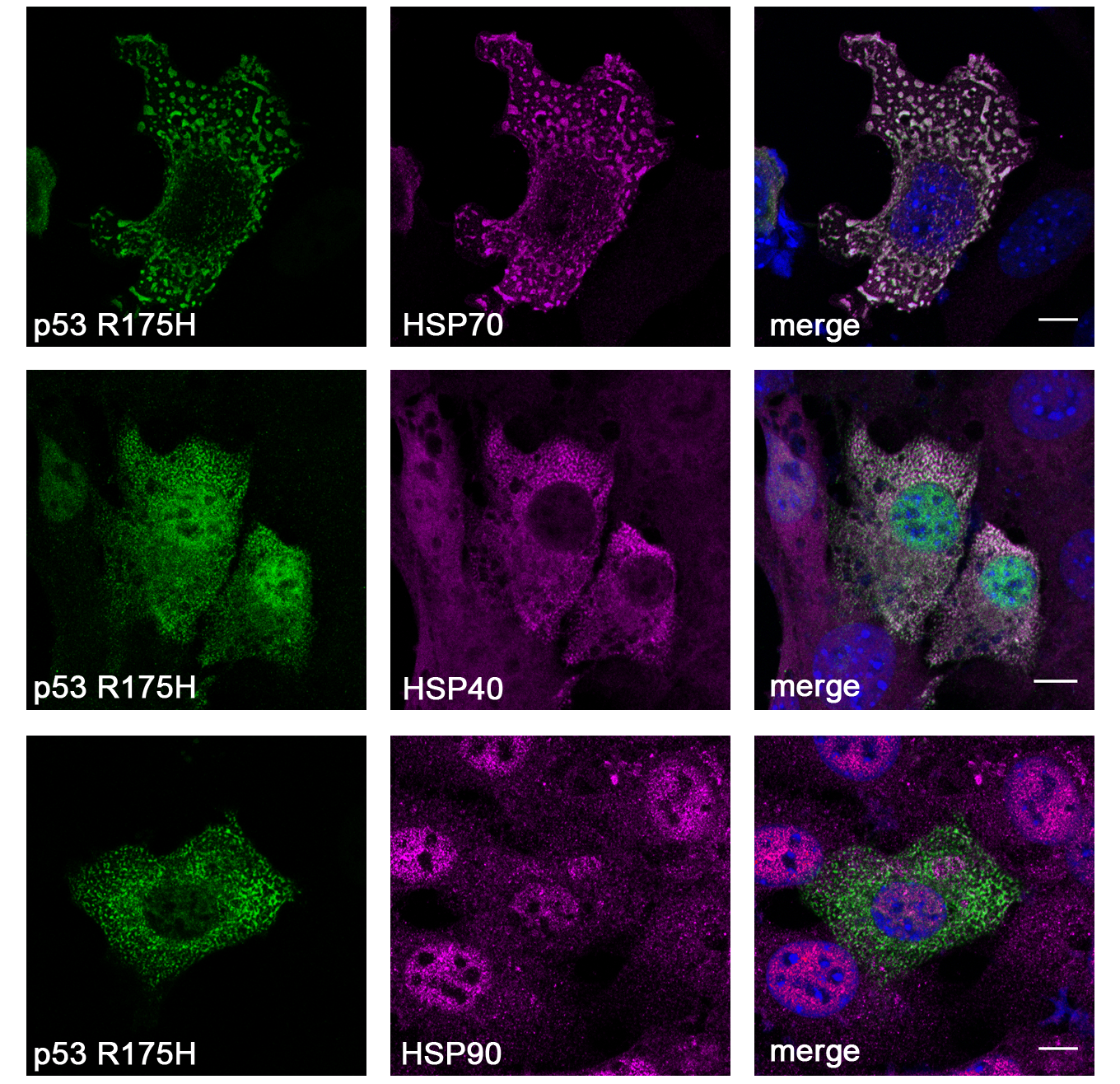

Supplement: Figure S6 — Composition of cytoplasmic p53 R175H protein spots. MEF cells were transfected with plasmids encoding p53 R175H and HSP70. 48 hours post-transfection the cells were fixed and labeled for mutant p53, HSP70, HSP40 and HSP90. Nucleus was stained with Hoechst (blue). Scale bar 10 µm. (TIF) [file pone.0051426.s006.tif]

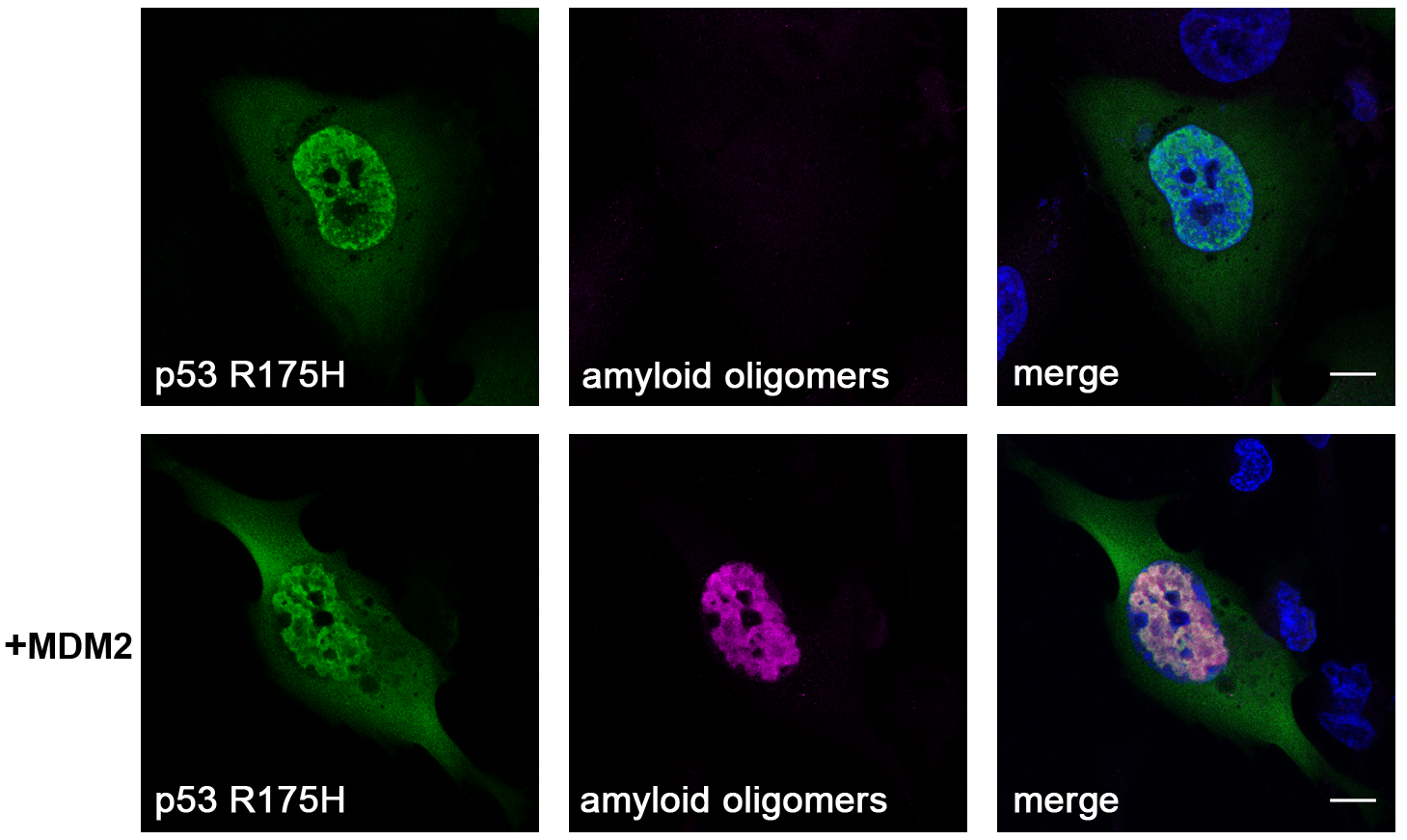

Supplement: Figure S7 — MDM2 induces amyloid-like aggregation of p53 R175H in the nucleus of H1299 cells. H1299 cells were transfected with plasmid encoding p53 R175H and optionally MDM2. 6 hours post-transfection medium was changed for one containing MG132 proteasome inhibitor (2 µM). 16 h later the cells were fixed and labeled for mutant p53 and amyloid oligomers. Nucleus was stained with Hoechst (blue). Scale bar 10 µm. (TIF) [file pone.0051426.s007.tif]

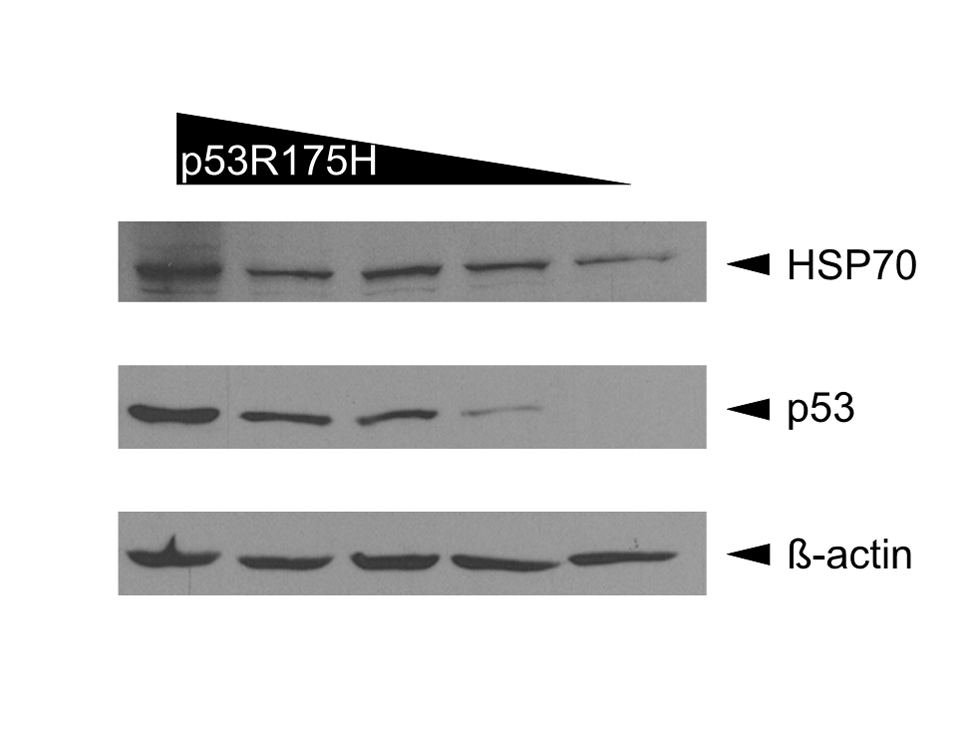

Supplement: Figure S8 — The level of HSP70 in MEFs increases upon overexpression of TP53 R175H. MEF cells were transfected with increasing amount of plasmid encoding p53 R175H. 24 hours post-transfection the cells were lysed and analyzed by western blotting. (TIF) [file pone.0051426.s008.tif]

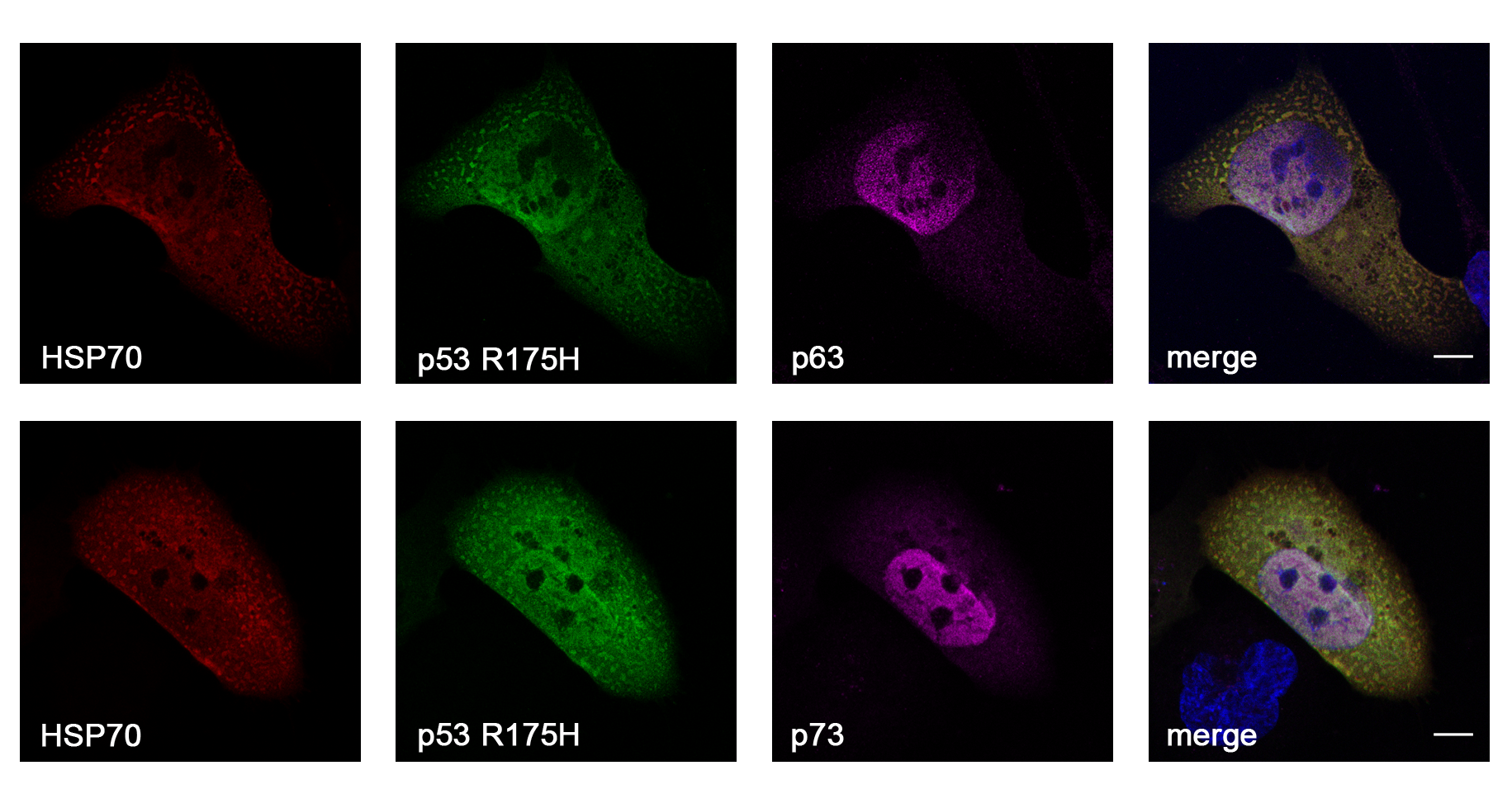

Supplement: Figure S9 — p53 R175H cytoplasmic pseudo-aggregates do not sequester transcription factors. H1299 cells were transfected with plasmids encoding p53 R175H and HSP70 parallel with either p63 or p73. 6 hours post-transfection medium was changed for one containing MG132 proteasome inhibitor (2 µM). 16 h later the cells were fixed and labeled for mutant p53, HSP70, p63 and p73. Nucleus was stained with Hoechst (blue). Scale bar 10 µm. (TIF) [file pone.0051426.s009.tif]
